# Synergistic chemodynamic and metabolic reprogramming-based cancer therapy by CuO@HA nanozymes with oxygen vacancy

**DOI:** 10.7150/thno.119806

**Published:** 2026-01-01

**Authors:** Weiwei Wang, Yuxuan Cai, Zhongxing Wang, Haoyang Song, Tangbin Hu, Ying Jia, Maoxiao Feng, Juyoung Yoon, Qiongzheng Hu, Yunshan Wang

**Affiliations:** 1 Department of Clinical Laboratory, Shandong Provincial Hospital Affiliated to Shandong First Medical University, Jinan, Shandong 250021, China.; 2 Qilu University of Technology (Shandong Academy of Sciences), Shandong Analysis and Test Center, Jinan, Shandong 250014, China.; 3 Department of Chemistry and Nanoscience, Ewha Womans University, Seoul 03760, Republic of Korea.; 4 Graduate Program in Innovative Biomaterials Convergence, Ewha Womans University, Seoul 03760, Republic of Korea.

**Keywords:** copper-based nanozymes, cancer therapy, metabolic reprogramming, tumor microenvironment

## Abstract

**Rationale:** The tumor microenvironment (TME), which is characterized by disordered metabolism, acidic pH, and high glutathione (GSH) and hydrogen peroxide (H_2_O_2_) levels, seriously hampers the efficacy of cancer therapy. Nanozymes with multi-enzyme activity have considerable potential to reprogram the TME and suppress tumor growth.

**Methods:** A strategy for remodulating the TME based on a hierarchical CuO nanozyme with oxygen vacancies decorated with hyaluronic acid (HA) (CuO@HA) was developed. The enzyme activities of CuO@HA were evaluated by enzyme kinetic assays and density functional theory (DFT). The *in vitro* and *in vivo* anti-tumor effects were estimated, and the mechanism was explored using multi-omic methods.

**Results:** CuO@HA exhibited effective peroxidase (POD)-like enzyme and glutathione oxidase (GSHOx)-like enzyme activities, which catalyzing the decomposition of H_2_O_2_ into toxic hydroxyl radicals (·OH) and oxidation of GSH into glutathione disulfide (GSSG), respectively. DFT calculations confirmed the effective catalytic activity of CuO@HA. Both *in vitro* and *in vivo* experiments demonstrated that CuO@HA can inhibit the growth of breast cancer and melanoma cells with no notable systemic toxicity by producing high levels of ·OH and reprogramming the glycine, serine, and threonine metabolism pathways in tumor tissues.

**Conclusions:** Our study is the first to demonstrate a strategy to reprogram the glycine, serine, and threonine metabolic pathways via the CuO nanoparticle-mediated downregulation of choline dehydrogenase (*Chdh*) in tumor tissues. This antitumor strategy, which combines chemodynamic therapy and reprogramming of amino-acid metabolism, represents a novel approach for cancer therapy.

## Introduction

Compared with normal tissues, tumor tissues are characterized by an immunosuppressive microenvironment, with disordered metabolism, higher glutathione (GSH) and hydrogen peroxide (H_2_O_2_) levels 1. The tumor microenvironment (TME) is one of the key factors responsible for the metastasis and invasion of tumors and failure of therapy 2, 3. Hence, strategies for remodeling the TME have been widely explored 4-7. Currently, relatively mature TME modulation strategies in clinical research commonly include immune checkpoint inhibition, anti-angiogenesis and vascular normalization, metabolic pathway intervention, and physicochemical factor regulation (such as pH, ROS, and temperature control). These methods typically have well-defined mechanisms of action and can substantially improve the TME in specific aspects; however, most face limitations such as single-target specificity, susceptibility to inducing drug resistance, or pronounced side effects 8, 9. In contrast, nanocatalyst-based TME modulation offers distinct advantages 10-13. Nanozymes combine the catalytic function of natural enzymes with the structural designability of nanomaterials, exhibiting multi-enzyme mimetic activity under typical tumor microenvironment conditions 14-17. Owing to the stable and controllable catalytic activities, physiochemical properties and low cost, nanomaterials with enzyme-like activity (nanozymes) have been developed for cancer diagnosis and therapy 18-24. Transition-metal-based nanozymes with empty d or f orbitals can form transition states with atoms with lower energy, contributing to efficient catalysis 25. Therefore, a series of transition-metal-based nanozymes, such as iron-, copper-, manganese-, cerium-, and noble metal-based nanozymes, have been developed 26-30. However, completely matching their catalytic efficiency and specificity to those of natural enzymes is challenging, and issues such as uncontrollable activity and limited long-term in vivo safety persist. The catalytic efficiency of nanozymes is paramount to their therapeutic outcome. Recently, defect engineering, particularly the introduction of oxygen vacancies, has emerged as a powerful strategy to enhance the enzyme-mimicking activities of nanomaterials 31-34. The profound biological consequences of this enhanced catalysis, particularly its impact on the metabolic networks of cancer cells, remain largely unexplored.

As a key co-factor of several metabolic enzymes, copper plays an important role in various biological activities and exerts unique regulatory effects 35, 36. Cu-based nanozymes possess a remarkable reaction rate for decomposing H_2_O_2_ to ·OH under flexible pH conditions mediated by a Fenton-like reaction, facilitating chemodynamic therapy 37, 38. In addition to enzyme-like activities, Cu-based nanozymes also exhibit the characteristics of nanomaterials, including ease of modification, prolonged circulation, and lower toxicity toward normal cells compared with copper ions 39. In the TME, Cu-based nanozymes can mimic multi-enzyme activities highlighting their immense potential for application in cancer therapy with minor side effects 40-42. Although Cu-based nanozymes have been studied for several years, a comprehensive understanding of their catalytic and anticancer mechanisms remains insufficient.

In recent years, nanotherapeutics based on metabolic reprogramming have rapidly emerged in the field of TME regulation. By combining their superior targeted delivery capabilities with metabolic intervention strategies, nanomaterials can precisely modulate metabolic pathways in tumor or immune cells. Amino acid metabolism within tumor tissues serves as a crucial nutritional source for tumor growth. Nanodrugs can inhibit tumor cell proliferation and improve the immune microenvironment by regulating amino acid metabolism. For example, certain nanocarriers can target specific amino acid metabolic pathways to inhibit tumor cell metabolism and enhance therapeutic efficacy 43, 44.

**Scheme 1:**
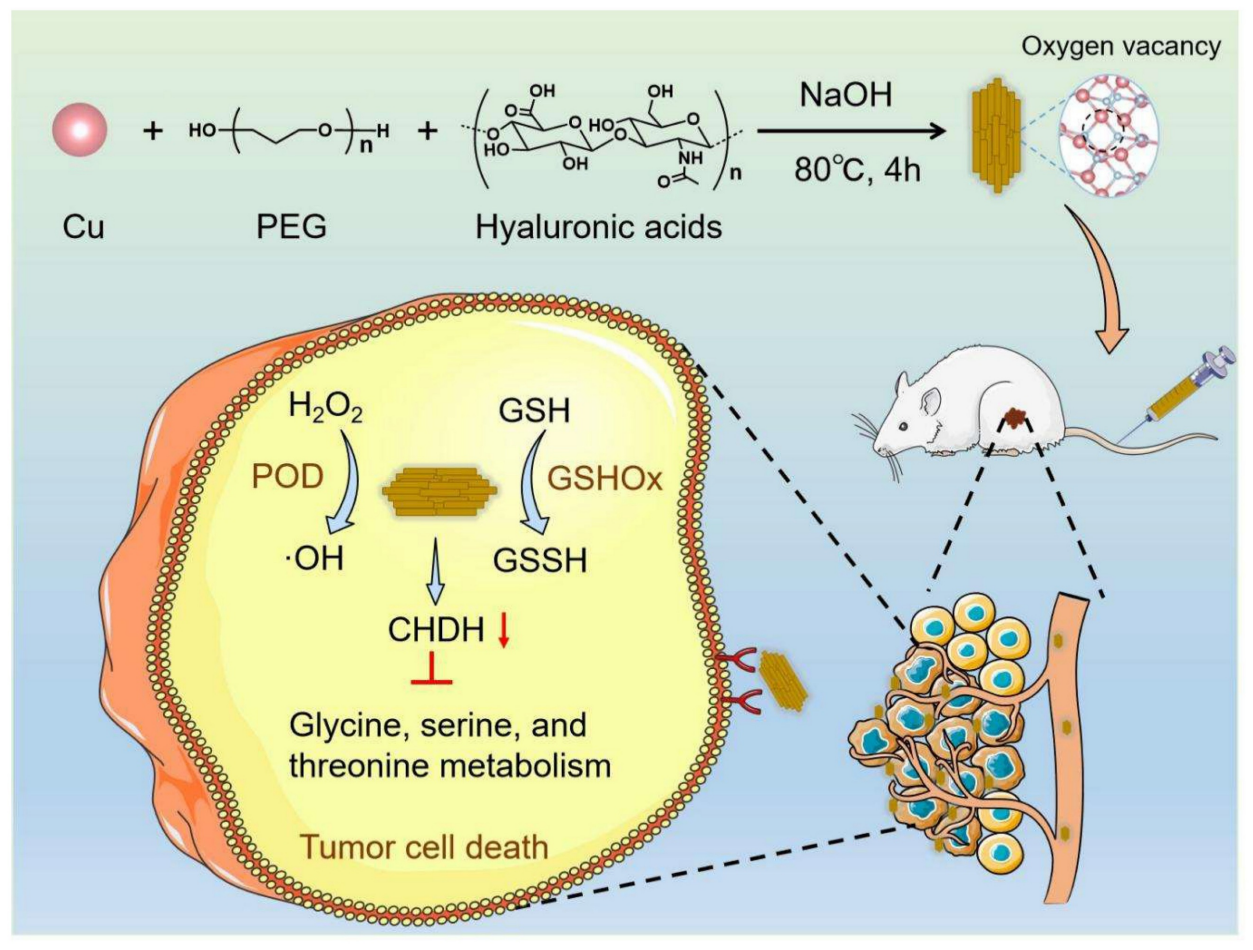
Schematic of the synthesis and antitumor mechanism of CuO@HA.

In the present study, CuO nanoenzymes were developed for reprogramming the TME. To improve biocompatibility and tumor targeting, hyaluronic acid (HA) was introduced to recognize CD44 receptors on tumor cells. The developed hierarchical CuO nanozyme with oxygen vacancies decorated with HA (CuO@HA) exhibited POD-like- and GSHOx-like enzyme activities. Density functional theory (DFT) calculations confirmed the highly efficient enzyme-like activity of CuO@HA. The multi-enzyme activities endowed CuO@HA with the capability to catalyze H_2_O_2_ into toxic ·OH, contributing to chemodynamic cancer therapy. Notably, CuO@HA reprogrammed the glycine, serine, and threonine metabolism pathways by downregulating choline dehydrogenase (Chdh), which disturbed the amino acid metabolism of tumors and promoted tumor cell apoptosis. Chdh, a specific enzyme located on the mitochondrial membrane, catalyzes the oxidation of choline to glycine betaine within the inner mitochondrial membrane. This process provides a methyl donor for the one-carbon unit/methyl cycle, thereby indirectly regulating the homeostasis of amino acids such as methionine. Within tumor cells, CHDH participates in the metabolic pathways of glycine, serine, and threonine 45, 46. Thus, the CuO@HA nanozyme with considerable biocompatibility and tumor-targeting ability suppressed tumor growth through synergistic therapy combining chemodynamic therapy and metabolic reprogramming (**Scheme 1**).

## Results and Discussion

### Synthesis and characterization of CuO@HA

**Figure 1:**
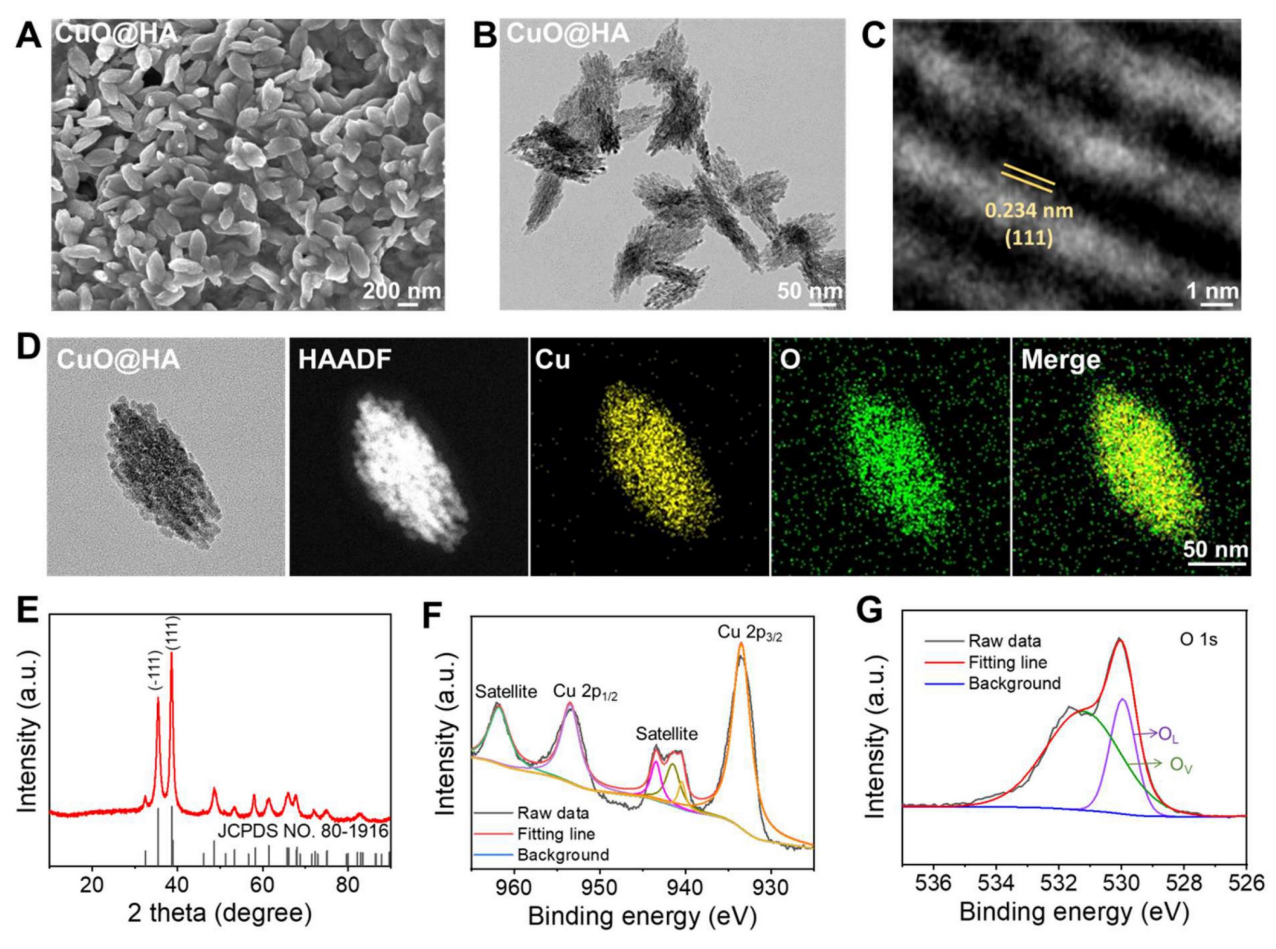
The characterization of nanoparticles. (**A**) SEM, (**B**) TEM, and (**C**) HR-TEM analyses of CuO@HA. (**D**) EDS-mapping of CuO@HA. (**E**) XRD of CuO@HA. XPS curves of (**F**) Cu 2p and (**G**) O 1s for CuO@HA (O_L_: lattice oxygen, O_V_: vacancy oxygen).

CuO@HA nanoparticles were prepared through a one-step method at 80℃ using copper acetate, HA, poly(ethylene glycol) (PEG) and sodium hydroxide (NaOH). Scanning electron microscopy (SEM) and transmission electron microscopy (TEM) revealed that CuO@HA with a uniform morphology had short, rod-like hierarchical nanoparticles (**Figure 1A-B**). High-resolution transmission electron microscopy (HRTEM) imaging of CuO@HA revealed clear lattice planes with a spacing of 0.234 nm, corresponding to the (111) plane of CuO (**Figure 1C**). Furthermore, according to the dynamic light scattering (DLS) results, the average hydrodynamic size of CuO@HA was 295 nm (**Figure S1**).

The elemental and chemical compositions of CuO@HA were determined using EDS mapping, X-ray diffraction (XRD), and X-ray photoelectron spectroscopy (XPS). EDS mapping showed a uniform distribution of Cu and O in CuO@HA (**Figure 1D**). The XRD peaks can be indexed to CuO (JCPDS No. 80-1916) (**Figure 1E**). XPS was performed to identify the composition and oxidation states of CuO@HA. The survey spectrum of CuO@HA confirmed the presence of Cu and O (**Figure S2**). The Cu 2p spectrum showed that the binding energies of Cu 2p_3/2_ and Cu 2p_1/2_ were 953.34 and 933.54 eV, respectively, and two satellites were observed at 961.75 and 943.42 eV, which are consistent with Cu^2+^ (**Figure 1F**). Besides, the O 1s spectrum showed two peaks at 529.9 eV and 531.3 eV corresponding to lattice oxygen and vacancy oxygen, respectively, and the oxygen vacancy was quantified as 28.23% by calculating the area ratio of the corresponding XPS spectral peaks (**Figure 1G**). To confirm the existence of oxygen vacancy, electron spin resonance (ESR) of CuO@HA was performed. The ESR spectrum of CuO@HA exhibited a broad signal with a *g* value of 2.101, attributed to unpaired electrons localized at oxygen vacancy sites in the CuO@HA (**Figure S3**). Hence, the results demonstrate that the Cu-based nanoparticles were CuO with oxygen vacancies, and the Cu species was Cu^2+^. CuO nanoparticles without HA were synthesized as controls to confirm the presence of HA in the CuO@HA. The SEM, TEM, and XRD results of CuO indicated that the crystal phase and morphology of CuO remained unchanged compared to those of CuO@HA (**Figure S4, Figure S5**). However, the DLS of CuO was 220 nm, smaller than that of CuO@HA, indicating an HA coating (**Figure S6**). After HA coating, the zeta potential of CuO shifted from 30.8 ± 1.1 to 19.7 ± 0.6 mV at pH 6.5 and from -6.9 ± 3.3 to -14.1 ± 0.5 at pH 7.4 (**Figure S7**). Notably, the surface charge of both CuO@HA and CuO reversed from positive at pH 6.5 to negative at pH 7.4. This charge-reversal property ensures the nanoparticle is negative during systemic circulation for stability and longevity but becomes positive upon reaching the acidic TME, which enhances tumor cell uptake and triggers copper ion release. Fourier-transform near-infrared spectroscopy (FT-IR) showed that the O-H stretching band of CuO@HA becomes broader than that of CuO. This broadening is a classic indication of the enhancement of hydrogen bonding networks, strongly suggesting that the hydroxyl groups of HA are involved in coordination with the copper ions on the surface of the CuO nanoparticles, forming Cu-O-H bonds. Most significantly, a new peak emerges at 1044 cm⁻¹ in the CuO@HA spectrum. The region is associated with C-O stretching vibrations (**Figure S8**).

Thermo-gravimetric analysis (TGA) was performed to measure the ratio of HA, PEG and CuO in CuO@HA. As depicted in **Figure S9**, a 8.9% weight loss of CuO@HA was observed, revealing 91.1% of Cu and 8.9% of HA and PEG. Notably, CuO@HA showed a 1.8% weight loss upon the HA modification compared to CuO, indicating the loading ratio of HA was 1.8% in CuO@HA. Therefore, the PEG content in CuO@HA was 7.1%. Considering the DLS, Zeta potential, FT-IR, and TGA results, the CuO NPs were successfully modified with HA.

### Multienzyme-like activities of CuO@HA

**Figure 2:**
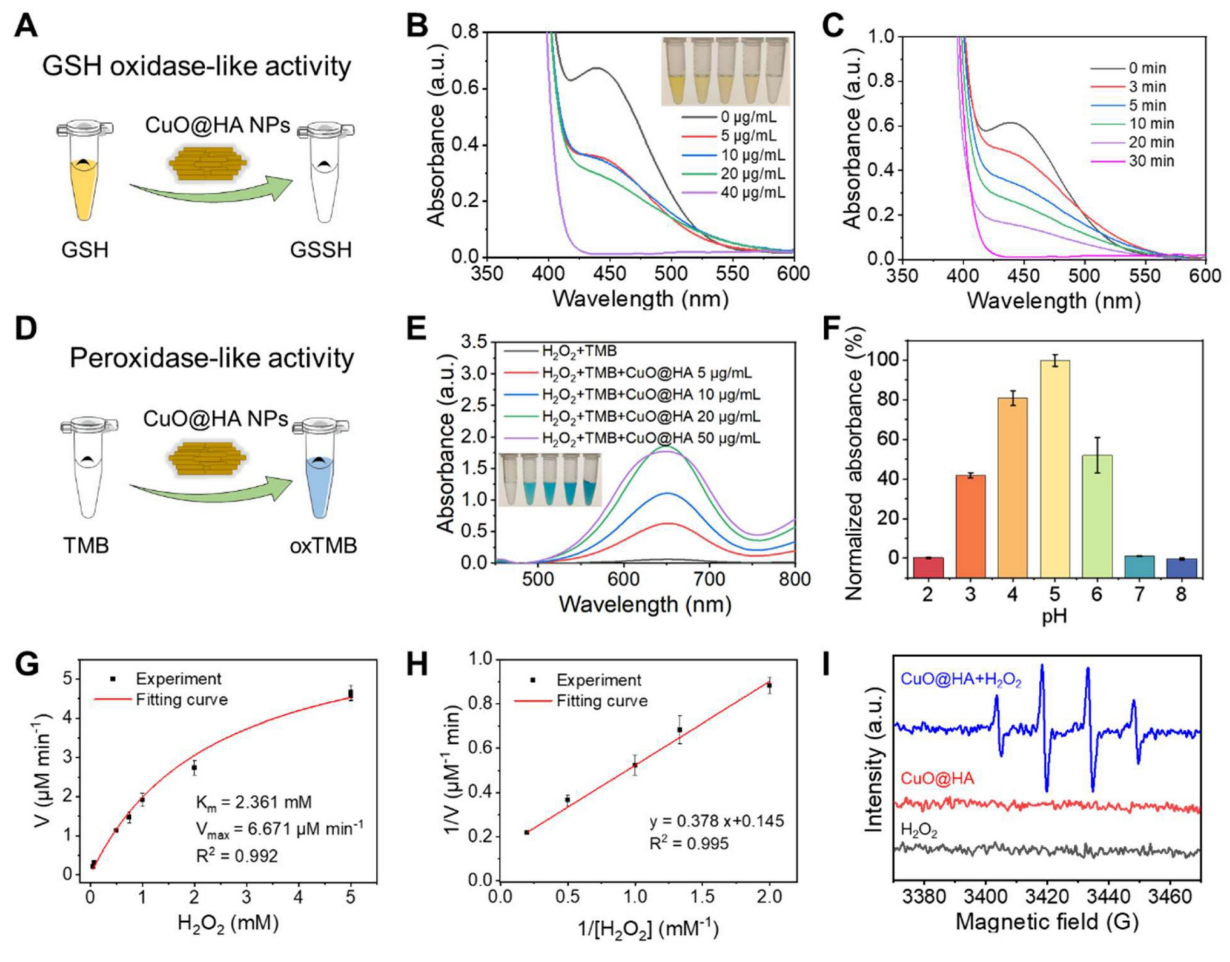
Enzyme-like activity characterization of CuO@HA. (**A**) Schematic of GSHOx-like activity of CuO@HA. UV-vis spectra of DTNB (**B**) treated with different concentrations of CuO@HA for 30 min (inset: the photo of DTNB color with different treatments) or (**C**) treated with 40 μg/mL CuO@HA for different time periods. (**D**) Schematic of the POD-like activity of CuO@HA. (**E**) UV-vis spectra of TMB under different conditions (inset: the photo of TMB color with different treatments). (**F**) The effect of pH on the absorbance of the TMB chromogenic reaction. (**G**) Michaelis-Menten fitting curves and (**H**) Lineweaver-Burke fitting curves for CuO@HA. (**I**) ESR spectra of ·OH trapped by 5,5-dimethyl-1-pyrroline N-oxide (DMPO) under different conditions. Data are presented as the mean ± SD (*n* = 3).

In this study, the ability of CuO@HA NPs to consume GSH (GSHOx-like enzyme) and decompose H_2_O_2_ to ·OH (POD-like enzyme) was explored. To determine GSH depletion, 5,5'-dithiobis-(2-nitrobenzoic acid) (DTNB), which reacts with GSH to produce a yellow product, was used as a probe (**Figure 2A**). As shown in **Figure 2B**, the characteristic UV-vis absorption decreased with an increase in the CuO@HA concentrations. At a CuO@HA concentration of 40 μg/mL, the characteristic peak weakened with time and disappeared at 30 min (**Figure 2C**). These results demonstrated that CuO@HA can effectively deplete GSH. The POD-like enzymatic activity of CuO@HA was assessed using 3,3',5,5'-tetramethylbenzidine (TMB). TMB was oxidized to a blue product with UV-vis absorption at 652 nm in the presence of H_2_O_2_ and POD (**Figure 2D**). The H_2_O_2_ + TMB + CuO@HA group showed a distinct blue color and absorption at 652 nm, illustrating the POD-like enzyme activity of CuO@HA (**Figure 2E, Figure S10**). The POD-like enzyme activity increased with an increase in CuO@HA concentration until 20 μg/mL (**Figure 2E**). In addition, the POD-like enzyme activity of CuO@HA was related to pH and temperature. At pH 5.0, CuO@HA showed higher POD-like enzyme activity than that at other pH values, which increased with increasing temperatures (**Figure 2F, Figure S11**). The POD-like enzyme activity kinetics of CuO@HA were analyzed. The Michaelis-Menten constant (K_M_) and the maximal reaction velocity (V_max_) were determined to be 2.361 mM and 6.671 μM/min, respectively, for the catalytic oxidation of the H_2_O_2_ substrate (**Figure 2G-H**). Furthermore, ESR was used to investigate ·OH production. As shown in **Figure 2I**, compared with H_2_O_2_ and CuO@HA, CuO@HA + H_2_O_2_ displayed distinct 1:2:2:1 ESR peaks, attributable to ·OH generation. Besides, a weak CAT-like activity was observed which could mitigate the hypoxic TME (**Figure S12**). To test the stability of CuO@HA, its enzymatic activity was measured after 90 days of storage. As shown in **Figure S13**, CuO@HA showed no significant reduction in enzyme-like activity after 90 d of storage. These results indicate that CuO@HA with stable GSHOx- and POD-like enzyme activity can eliminate GSH and decompose H_2_O_2_ to ·OH.

**Figure 3:**
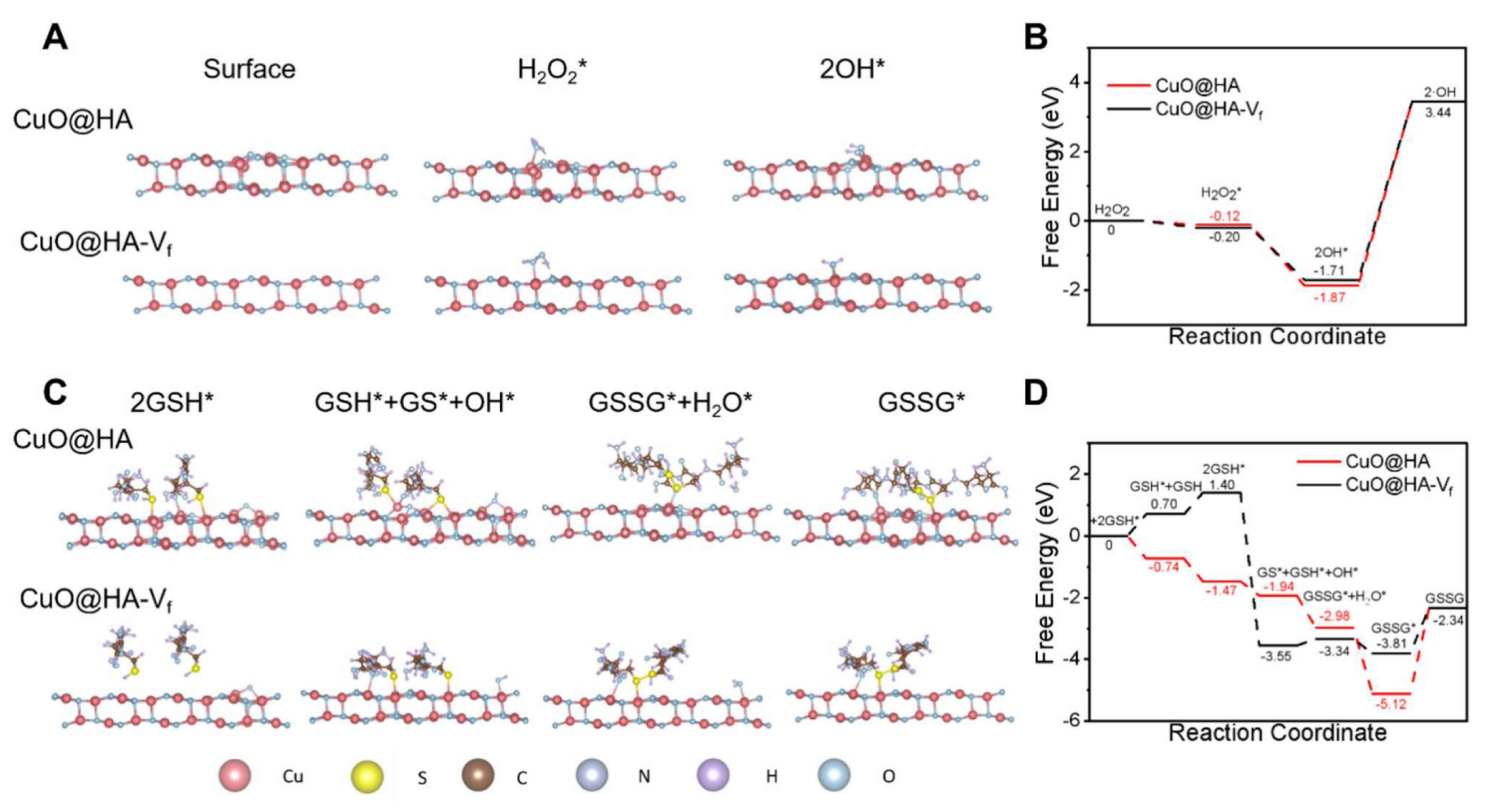
DFT calculations of POD-like activity (**A, B**) and GSHOx-like activity (**C, D**). The catalytic mechanisms (**A, C**) and free energy diagrams (**B, D**) of CuO@HA with vacancy (CuO@HA) and vacancy-free CuO@HA (CuO@HA-V_f_).

Further, DFT calculations were performed to understand the catalytic mechanism of CuO@HA and vacancy-free CuO@HA (CuO@HA-V_f_) was performed as a control. For POD-like activity, the H_2_O_2_ molecules adsorbed on nanozymes surface. Subsequently, the H_2_O_2_* molecules were dissociated into two hydroxyl group (OH*) and detached to form ·OH (**Figure 3A-B**). The H_2_O_2_ molecules were preferentially absorbed on the Cu atoms around the oxygen vacancy of CuO@HA with a calculated adsorption energy of -0.12 eV which higher than CuO@HA-V_f_ (-0.20 eV), indicating a stronger binding affinity of CuO@HA than of CuO@HA-V_f_. Therefore, CuO@HA exhibited highly efficient H_2_O_2_ adsorption and POD-like catalytic performance, and the oxygen vacancy led to an efficient adsorption and dissociation of H_2_O_2_. For the GSHOx-like activity, GSH molecules were absorbed on Cu atoms (GSH*) and reacted to form GS* and OH*. Obviously, GSH was adsorbed on CuO@HA-V_f_ through a heat absorption process while it was adsorbed on CuO@HA through an exothermic process, demonstrating that CuO@HA was more favorable to the adsorption of GSH than CuO@HA-V_f_. Subsequently, OH* reacted with GSH* to generate GSSG* and H_2_O. Notably, the energy barrier for CuO@HA-V_f_ was -3.55 eV to -3.44 eV. The results highlighted the considerable GSHOx-like enzyme activity of CuO@HA and showed that the introduction of oxygen vacancy lowered the energy barrier (**Figure 3C-D**).

### *In vitro* antitumor effects of CuO@HA

**Figure 4:**
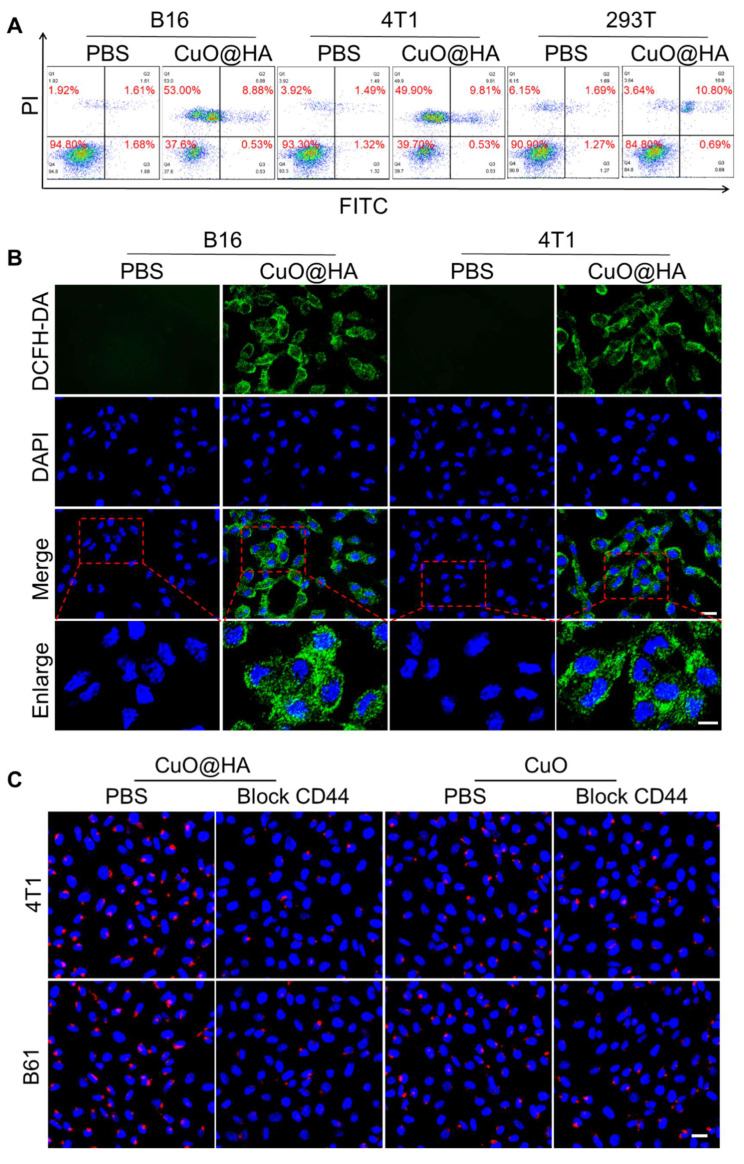
(**A**) Flow cytometric analysis of apoptosis in B16 and 4T1 cells cultured with 40 μg/mL CuO@HA for 12 h. (**B**) ROS levels of B16 and 4T1 cells cultured with PBS or 40 μg/mL CuO@HA for 12 h. Scale bar, 20 μm and 10 μm for enlarge. (**C**) Confocal imaging of 4T1 cells, 4T1 cells pre-treated with HA (block CD44), B16 cells or B16 cells pre-treated with HA after co-culturing with CuO@HA-Cy5.5 or CuO-Cy5.5. Scale bars, 20 μm.

The stability of CuO@HA in PBS and RPMI-1640 culture medium was assessed via DLS. As shown in **Figure S14**, the size of CuO@HA displayed no significant change after incubation with PBS or RPMI-1640 culture medium for 24 h, indicating high stability. The apoptosis of tumor cells and biocompatibility of CuO@HA were evaluated. The Cu release in GSH or HAase at different pH values was investigated. At pH 7.4, 5.1 ± 1.0%, 26.2 ± 1.5% and 8.6 ± 1.3% of Cu was released in PBS, GSH and HAase, respectively, and a higher concentration of Cu were released at pH 6.5 (17.7 ± 1.9% for PBS, 32.6 ± 1.5% for GSH and 22.5 ± 1.5% for HAase) (**Figure S15**). The results showed that CuO@HA exhibits stability at pH 7.4 in PBS or at pH 7.4 in HAase, with minimal copper ion leakage. In contrast, a pronounced release of Cu was triggered under acidic environments or under high GSH levels, demonstrating the biocompatibility and tumor microenvironment response ability of CuO@HA. The CCK-8 results indicated that the viabilities of B16 and 4T1 cells were reduced to 20.92 ± 3.75% and 20.14 ± 3.55%, respectively, when the concentration of CuO@HA was 40 μg/mL (**Figure S16**). In contrast, the viability of 293T cells remained at 80.77 ± 4.04%, even at a CuO@HA concentration as high as 100 μg/mL. In addition, compared with CuO@HA, CuO exhibited lower biocompatibility. The 293T cell viability was only 65.95 ± 3.23% at a CuO concentration of 100 μg/mL. Besides, *in vitro* hemolysis experiments were performed to assess the potential toxicity of CuO and CuO@HA. Compared with the negative control (PBS), CuO@HA maintained a colorless supernatant even at a high concentration of 200 μg/mL. Notably, CuO triggered mild hemolysis at a much lower concentration (10 μg/mL) (**Figure S17**). Hence, the introduction of HA during the synthesis resulted in CuO with high biocompatibility. The ability of CuO@HA to kill tumor cells was confirmed using flow cytometry and live/dead cell staining. Flow cytometry analysis revealed apoptosis of the B16 and 4T1 cells after treatment with CuO@HA (**Figure 4A**). Calcein-AM/PI staining showed that the tumor cells treated with CuO@HA underwent more cell death than those treated with PBS (**Figure S18**). The ROS in B16 and 4T1 cells induced by CuO@HA were studied using 2',7'-dichlorodihydrofluorescein diacetate (DCFH-DA) as a probe. As shown in confocal images and flow cytometry (**Figure 4B, Figure S19, Figure S20**), an increase in fluorescence intensity was observed after co-culturing with CuO@HA, indicating a high level of ROS production. Moreover, the GSH concentration in B16 or 4T1 cells after treatment with CuO@HA was measured by enzyme-linked immunosorbent assay. As shown in **Figure S21**, with an increase in the CuO@HA concentration, the GSH contents in B16 or 4T1 cells significant reduced, demonstrating the GSH-scavenging ability of CuO@HA at cellular level. To establish CD44-mediated tumor targeting with greater certainty, CuO@HA and CuO modified with Cy5.5 (CuO@HA-Cy5.5 and CuO-Cy5.5) were built to monitor the dynamic of nanoparticles *in vitro.* Fluorescence scans were performed to confirm the modification. A significant emission peak was observed at 707 nm for CuO@HA-Cy5.5 and CuO-Cy5.5 (exitation at 680 nm), demonstrating the successful modification of Cy5.5 (**Figure S22**). Subsequently, CuO@HA-Cy5.5 and CuO-Cy5.5 were co-cultured with 4T1 cells, B16 cells, 4T1 cells pre-treated with HA, and B16 cells pre-treated with HA. According to the confocal imaging and the gray value statistics, 4T1 and B16 tumor cells absorbed 1.59-fold and 1.47-fold more CuO@HA than CuO, respectively (**Figure 4C, Figure S23**). After pre-treatment with free HA, tumor cells showed reduced CuO@HA uptake compared with untreated cells, confirming CD44-dependent cellular uptake. These results indicate that CuO@HA with great CD44-mediated tumor target ability promoted ROS production and GSH depletion, leading to tumor cell death.

### *In vivo* antitumor effect of CuO@HA

**Figure 5:**
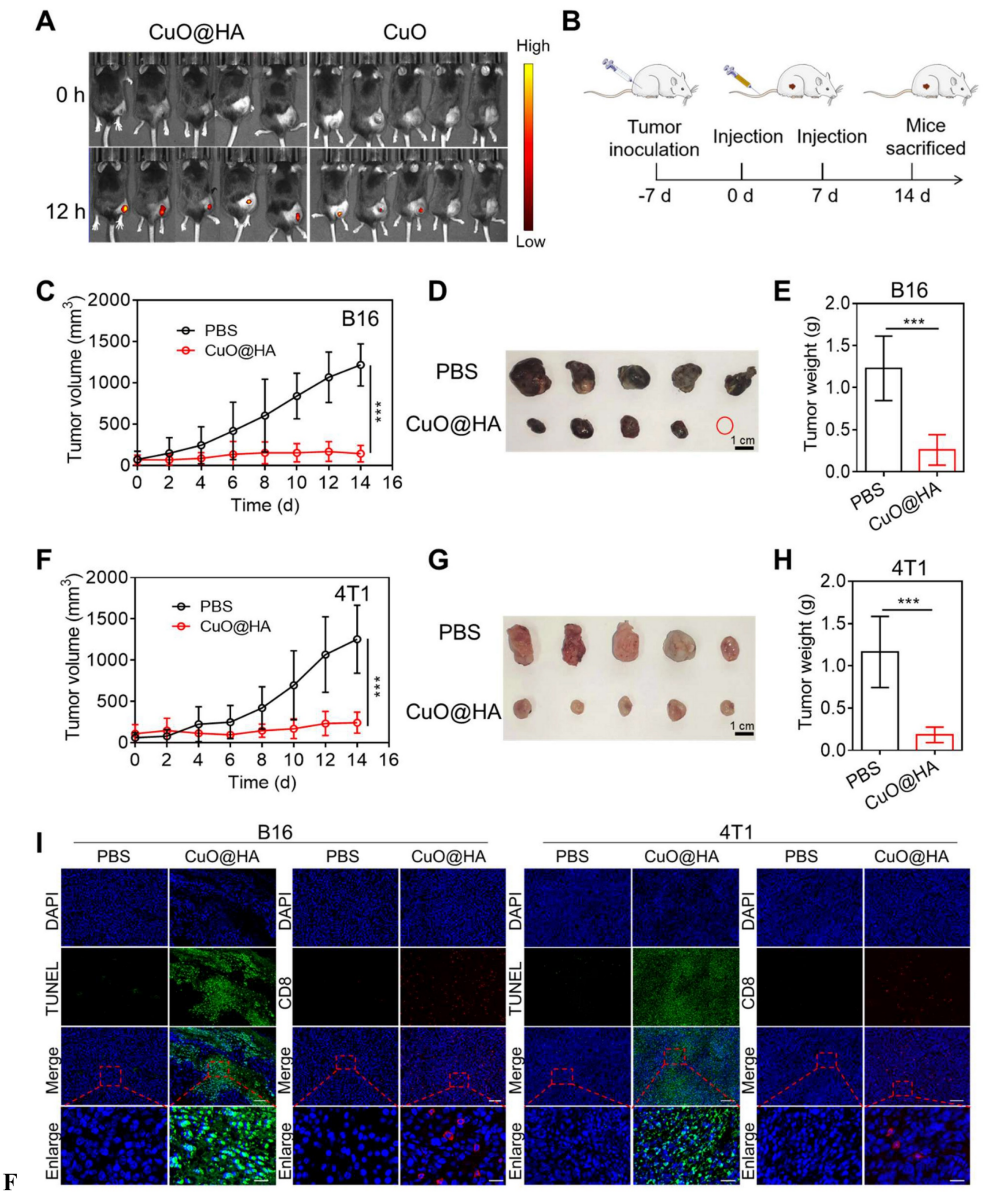
(**A**) Fluorescence imaging of B16 tumor-bearing mice after intravenous injection with CuO@HA-Cy5.5 or CuO-Cy5.5 at 0 and 24 h. (**B**) Scheme of the anticancer experiment design. (**C-E**) B16 tumor therapeutic effect. (**C**) Tumor growth curves of the mice during therapy. (**D**) Photographs and (**E**) weights of tumor tissues. (**F, H**) Therapeutic effects in 4T1 cells. (**F**) Tumor growth curves of the mice during therapy. (**G**) Photographs and (**H**) weights of tumor tissues. (**I**) H&E, Ki67, TUNEL, and CD8 T staining of B16 and 4T1 tumor tissues after different treatments. Scale bar, 100 μm for H&E, 500 μm for Ki67, 100 μm for TUNEL, 100 μm for CD8 T and 20 μm for enlarge. Data are presented as the mean ± SD (*n* = 5). *p < 0.05, **p < 0.01, and ***p < 0.001.

Owing to its enzyme-like activities and toxicity to tumor cells, CuO@HA was expected to exert an excellent antitumor effect *in vivo*. To establish CD44-mediated tumor targeting with greater certainty, B16 tumor-bearing mice were intravenously injected with CuO@HA-Cy5.5 or CuO-Cy5.5 for *in vivo* fluorescence imaging at 0 and 24 h, and the organs and tumors were collected for fluorescence imaging at 24 h. Based on the results, the mice injected with CuO@HA showed higher fluorescence intensity in the tumor than in the CuO group (**Figure 4A, Figure S24**). Besides, the tumor-targeting ability and distribution of CuO@HA and CuO were analyzed at 0, 12, and 24 h post injection. The Cu concentration in the major organs and tumor tissues of 4T1 tumor-bearing mice after the intravenous injection of CuO@HA or CuO was detected via inductively coupled plasma-mass spectrometry (ICP-MS) (**Figure S25**). In the CuO@HA group, a high concentration of Cu was observed in the liver, followed by that in the tumor, illustrating the accumulation of CuO@HA in the liver and tumors. In contrast, CuO was mostly enriched in the liver and spleen and minimally accumulated in the tumor. Hence, CuO@HA showed excellent accumulation in the tumor, which was attributed to the HA modification. The antitumor effects were systematically evaluated *in vivo*. B16 tumor-bearing mice were intravenously injected with CuO@HA twice, and tumor growth was monitored (**Figure 5B**). The tumor growth curves showed notable inhibition of tumor development in the CuO@HA group. The tumor volume of the PBS group steadily increased and reached 1215 ± 254 mm^3^ at 14 d after the first injection, whereas the tumor volume of the CuO@HA group was restrained to only 142 ± 99 mm^3^ at 14 d (**Figure 5C-E, Figure S26**). In addition, a 4T1 tumor model was established to further confirm the universality of CuO@HA tumor therapy, and similar results were obtained (**Figure 5F-H, Figure S27**). Furthermore, the necrosis and cell proliferation in tumor tissues were detected using hematoxylin and eosin (H&E), terminal deoxynucleotidyl transferase dUTP nick end labeling (TUNEL), and Ki67 staining. H&E and TUNEL staining revealed severe cell necrosis and apoptosis in the B16 and 4T1 tumor tissues of the mice treated with CuO@HA (**Figure 5I, Figure S28**). Ki67 staining revealed that CuO@HA decreased the cell proliferation to a level lower than that observed with PBS. Furthermore, the infiltration of CD8^+^ T cells into tumor tissues was investigated by immunofluorescence staining. The results indicated considerable infiltration of CD8^+^ T cells into the B16 or 4T1 tumor tissues of the CuO@HA treatment group. No obvious body weight loss was observed during therapy (**Figure S29**). Blood biochemistry assays and H&E staining of the major organs showed no visible damage to the mice after CuO@HA treatment (**Figure S30**). Based on these results, CuO@HA has an outstanding antitumor effect and good biosafety.

### Mechanisms underlying the antitumor activity of CuO@HA

**Figure 6:**
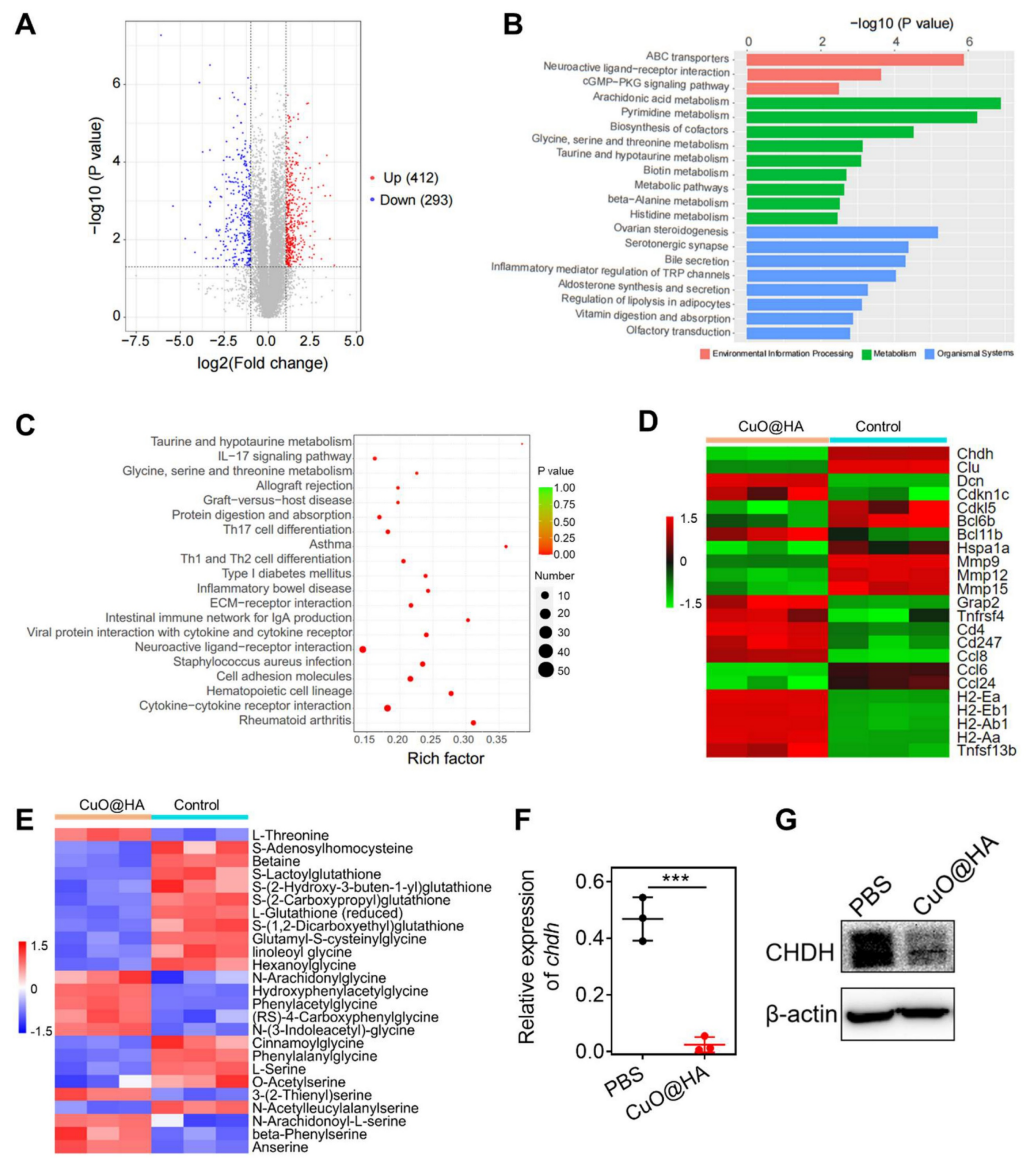
(**A**) Volcano plot of upregulated and downregulated metabolism. p < 0.05, Fold change (FC) > 2. (**B**) KEGG enrichment analysis of non-target metabolomics. (**C**) KEGG enrichment analysis of RNA-seq. (**D**) Heatmap of differentially expressed gene. (**E**) Heatmap of differentially expressed metabolism. (**F, G**) Chdh expression in 4T1 cells treated with PBS or CuO@HA measured by qRT-PCR (**F**) and WB (**G**). Data are presented as the mean ± SD (*n* = 3). *p < 0.05, **p < 0.01, and ***p < 0.001.

To explore the antitumor mechanism of CuO@HA, 4T1 tumor tissues from mice treated with CuO@HA or PBS were collected for non-target metabolomics and RNA-seq. In total, 412 upregulated and 293 downregulated metabolites were detected after CuO@HA treatment (**Figure 6A, Figure S31**). Kyoto Encyclopedia of Genes and Genomes (KEGG) enrichment analysis showed that the differentially expressed metabolites were mainly enriched in amino acid metabolism, such as glycine, serine, and threonine metabolic; taurine and hypotaurine metabolic; beta-alanine metabolic; and histidine metabolic pathways (**Figure 6B**). Recently, interfering with tumor metabolism has become a prominent method for cancer therapy 47-49. Amino acid metabolism in tumor tissues, which supplies cell building blocks and energy for tumor growth, is a key pathway for providing nutrition to tumor cells 50, 51. Reprogramming amino acid metabolism is considered a potential anticancer strategy 52-54. Furthermore, RNA-seq was performed to further investigate the molecular mechanism underlying the effects of CuO@HA. KEGG enrichment analyses revealed significant alterations in the glycine, serine, and threonine metabolism pathways, which is consistent with the results of non-target metabolomics. The glycine, serine, and threonine metabolism axes participate in one-carbon metabolism and the folate cycle, which are crucial for tumor cell survival and proliferation 55, 56. Regulating the metabolism of glycine, serine, and threonine in tumors may be beneficial for cancer inhibition 50. Besides, a series of immune-related pathways was altered, such as the IL-17 signaling pathway, Th17 cell differentiation, Th1 and Th2 cell differentiation, and cytokine-cytokine receptor interactions (**Figure 6C**). Notably, 1411 genes were involved, among which 1017 were upregulated and 394 were downregulated (**Figure S32, Figure S33**). According to **Figure 6D**, CHDH, a mitochondrial enzyme that participates in the glycine, serine, and threonine metabolic pathways, was significantly downregulated. The downregulation of matrix metalloproteinases (*Mmp9*, *Mmp12*, *Mmp15*), which are enzymes that degrade the extracellular matrix to allow tumor invasion and metastasis, indicating a severe impairment of the tumor invasive capacity. And the downregulation of *Clu*, a potent anti-apoptotic protein, and *Bcl6b*, a transcriptional repressor involved in cell survival, demonstrates tumor cells more susceptible to apoptosis. The decrease in *Hspa1a*, a heat shock protein that protects cells from various stresses, suggests an oxidative stress induced by CuO@HA.

Besides, a series of genes essential for antigen presentation and T cells activation were upregulated after CuO@HA treatment. The increase in major histocompatibility complex class II genes (*H2-Ea*, *H2-Eb1*, *H2-Ab1*, *H2-Aa*) indicates that CuO@HA treatment enables tumor cells or antigen-presenting cells to more effectively display tumor antigens, which is the critical first step in activating the immune system. The upregulation of *Ccl8* (a chemokine that recruits T-cells and other immune cells) and *Cd4* (a marker for helper T-cells) suggests active recruitment and infiltration of immune cells into the tumor. Furthermore, the increase in *Tnfrsf4* and *Tnfsf13b*, key costimulatory molecules, and *Cd247*, a central component of the T-cell receptor, points to a robust activation of T-cell-mediated immunity. In summary, the RNA-seq data reveals a comprehensive explanation for the potent anti-tumor efficacy of CuO@HA linking metabolic reprogramming to intrinsic vulnerability and immune activation. A heat map of the differentially expressed metabolites is shown in **Figure 6E** to elaborate the disturbance of amino acids metabolism induced by CuO@HA mediated *Chdh* downregulation.

The significant downregulation of Betaine confirms the inhibition of *Chdh* enzyme activity, as Betaine is the direct product of the *Chdh*-catalyzed reaction. The decrease in S-Adenosylhomocysteine (SAH) indicates an impairment of cellular methylation capacity, a consequence of dysfunctional one-carbon metabolism. The downregulation of L-Serine and O-Acetylserine provides direct evidence of the exhaustion of central amino acid pools. The accumulation of L-Threonine (a precursor to glycine synthesis) indicates the cells attempt to ramp up glycine production via alternative pathways, underscoring the severe shortage of glycine downstream. The upregulation of a wide array of glycine-conjugated metabolites (N-Arachidonylglycine, Hydroxyphenylacetylglycine, Phenylacetylglycine, (RS)-4-Carboxyphenylglycine, N-(3-Indoleacetyl)-glycine, linoleoyl glycine) is an evidence of cellular glycine deficiency. Similarly, the upregulation of various serine derivatives (3-(2-Thienyl)serine, N-Arachidonoyl-L-serine, beta-Phenylserine) points to a dysregulation of serine metabolism. This metabolomic evidence validating the central role of *Chdh* downregulation. To confirm the effects of CuO@HA on Chdh expression, reverse transcription polymerase chain reaction (RT-PCR) and western blotting (WB) were performed. According to the results, the CHDH expression in CuO@HA-treated 4T1 cells was lower than that in PBS-treated cells (**Figure 6F-G**). Thus, the non-target metabolomics and RNA-seq results indicated that CuO@HA reprogrammed amino acid metabolism and the immune microenvironment of the tumor tissue, thereby inhibiting cancer development.

## Conclusions

In summary, we developed an effective method to reprogram the TME for cancer therapy using CuO@HA and systematically investigated the underlying mechanisms. CuO@HA accumulates in tumor tissues effectively after intravenous injection owing to the decoration of HA, which can be recognized by the CD44 receptor of tumor cells. Owing to the high levels of GSH and H_2_O_2_ in tumor tissues, CuO@HA exerts multiple enzyme-like activities, including POD-like- and GSHOx-like enzyme activities. The effective enzyme activities of CuO@HA catalyze the H_2_O_2_ into toxic ·OH and deplete GSH. Through multiomics analyses, we observed that after treatment with CuO@HA, the glycine, serine, and threonine metabolic pathways were reprogrammed by downregulation of *Chdh* expression in tumor tissues. Taken together, CuO@HA with dual enzyme activity inhibited tumor growth through tumor nanocatalytic therapy and the modulation of amino acid metabolism, opening a novel horizon for cancer therapy.

## Supplementary Material

Supplementary figures and table.
