# Supplementary material for: Synergistic chemodynamic and metabolic reprogramming-based cancer therapy by CuO@HA nanozymes with oxygen vacancy
Source: Theranostics. 2026 Jan 1;16(3):1374–85. doi: 10.7150/thno.119806 (PMC12679366; doi:10.7150/thno.119806)
Supplement: Supplementary file 1 — Supplementary figures and table. [file thnov16p1374s1.pdf]

Supporting Information

Synergistic chemodynamic and metabolic reprogramming-based cancer therapy by CuO@HA nanozymes with oxygen vacancy

Weiwei Wang [1,#], Yuxuan Cai [1,#], Zhongxing Wang [2], Haoyang Song [3,4], Tangbin Hu [1], Ying Jia [1], Maoxiao Feng [1], Juyoung Yoon [3,4,], Qiongzheng Hu [2,*] and Yunshan Wang [1,*]*

[1]Department of Clinical Laboratory, Shandong Provincial Hospital Affiliated to Shandong First Medical University, Jinan, Shandong 250021, China.

[2]Qilu University of Technology (Shandong Academy of Sciences), Shandong Analysis and Test Center, Jinan, Shandong 250014, China.

[3]Department of Chemistry and Nanoscience, Ewha Womans University, Seoul 03760, Republic of Korea

[4]Graduate Program in Innovative Biomaterials Convergence, Ewha Womans University, Seoul 03760, Republic of Korea

[*]**Corresponding Authors:**

E-mail: jyoon@ewha.ac.kr (J. Yoon); huqz@qlu.edu.cn (Q. Hu); wangyunshansd@sdu.edu.cn (Y. Wang).

[#]The authors contributed equally to this paper.

1. Supplementary materials

Cupric acetate anhydrous, poly(ethylene glycol) (PEG) and hyaluronic acid (HA, 40-100 kDa) were purchased from Macklin Biochemical Technology Co., Ltd. (Shanghai, China). Sodium hydroxide (NaOH) and hydrogen peroxide (H_2O_2) were purchased from Sinopharm Chemical Reagent Co., Ltd. (Shanghai, China). 3,3',5,5'-Tetramethylbenzidine (TMB) was purchased from Energy Chemical (Shanghai, China). Glutathione (GSH) was purchased from Aladdin Biochemical Technology Co., Ltd. (Shanghai, China). 2',7'-Dichlorodihydrofluorescein diacetate (DCFH-DA) was purchased from Solarbio Science & Technology Co., Ltd. (Beijing, China). Hoechst 33342 was purchased from Servicebio Technology Co., Ltd. (Wuhan, China). Fetal bovine serum (FBS), RPMI-1640 medium, DMEM medium, 0.25% Trypsin-EDTA and Penicillin-Streptomycin were purchased from Gibco Life Technologies (Grand Island, NY, USA). Cell counting kit-8 (CCK-8), GSH assay kit and Calcein/PI cell viability/cytotoxicity assay kit were obtained from Beyotime Biotechnology (Shanghai, China). The deionized water was obtained by a Milli-Q device (18.2 MΩ, Millipore). All chemical reagents were used without further purification.

2. Supplementary Methods

2.1. Synthesis of CuO@HA nanoparticles

Copper acetate (30 mg) was dissolved in 5 mL of deionized water under continuous magnetic stirring (500 rpm) at room temperature to form a homogeneous blue solution. Then, 200 μL of PEG (100 mg/mL, MW 3500 Da) and 100 μL hyaluronic acid (2.5 mg/mL, MW 40-100 kDa) were added. The mixture was stirred for an additional 5 min to ensure complete dispersion. The reaction system was heated to 80 °C under stirring (500 rpm). After thermal equilibration, 200 μL NaOH (40 mg/mL) was dropwise added at a rate of 10 μL/s. The reaction was allowed to proceed for 4 h at 80°C to ensure complete growth and crystallinity of the nanoparticles. The resulting CuO@HA nanoparticles were then collected by centrifugation (5000 rpm, 3 min) and washed with deionized water for three times.

2.2. Evaluation of POD-like enzyme activity

The POD-like enzyme activity of nanoparticles was measured using TMB as a probe. In brief, H_2O_2 (1mM), TMB (1mM), H_2O_2 + TMB, or H_2O_2 + TMB + different concentration of nanoparticles were dispersed in PBS (pH 5.0), respectively. After 5 min, the absorbance of solutions were recorded by a UV-visible spectrophotometer. The ·OH generation of CuO@HA was analyzed by ESR with DMPO as the spin trap via a same method.

2.3. Evaluation of GSHOx-like enzyme activity

The GSHOx-like enzyme activity of CuO@HA NPs or CuO NPs were evaluated by UV-visible spectrophotometer using DTNB as a probe. In detail, GSH (1 mM) mixed with nanoparticles for 30 min at 37°C, and then 50 μL of the mixture was added to 450 μL DTNB (2 mM) at pH 8.0. The absorbance was measured by UV-visible spectrophotometer.

2.3. Evaluation of CAT-like enzyme activity

The CAT-like activity of CuO@HA nanozyme by the dissolved oxygen meter. In detail, different concentration of CuO@HA (0, 5, 10, 20, 50 μg/mL) were mixed with H_2O_2 (5 mM) in 5 mL PBS (pH 6.5). The generation of O_2 was detected at different reaction time.

2.4. Cell culture

The 4T1 cell lines, B16 cell lines and 293T cell lines were purchased from OriCell (Guangzhou, China). 4T1 cells and B16 cells were cultured in RPMI-1640 medium containing 10% FBS and 1% antibiotics. 293T cells was cultured in DMEM medium containing 10% FBS and 1% antibiotics. All cells were cultured at 37°C in 5% CO_2.

2.5. Cytotoxic assay

The cytotoxicities of CuO@HA NPs or CuO NPs were measured by CCK-8 reagent. 4T1 cells, B16 cells or 293T cells were seeded in 96-well plates and incubated with nanoparticles at different concentration (0, 5, 10, 20, 40, 80, 100 μg/mL) for 12 h. And then 10 μL of CCK-8 was added and incubated for another 1 h at 37°C. Finally, a microplate reader was used to detect the absorption at 450 nm and calculate cell viability.

2.6. Live/Dead cell staining

Calcein/PI cell viability/cytotoxicity assay kit was used to assess the cell viability

through confocal imaging. 4T1 cells or B16 cells were seeded in confocal dishes and incubated with PBS or CuO@HA NPs (40 μg/mL) for 12 h. The cells were washed with PBS and stained with calcein AM and PI for 30 min at 37°C. Then the fluorescence of cells were observed by confocal microscopy.

2.7. Flow cytometry detection of cell apoptosis

4T1 cells, B16 cells or 293T cells were seeded in 6-well plates and co-cultured with PBS or CuO@HA (40 μg/mL). After 12 h, the single-cell suspension were stained with Annexin-FITC/PI and then analyzed by flow cytometry.

2.8 Hemolysis assay

The hemolysis assay was performed to assess in vitro biocompatibility. In detail, fresh mouse blood was collected and anticoagulated with 0.2 mL of heparin. The blood was diluted with 0.9% saline and centrifuged at 5000 rpm for 5 min to isolate the red blood cells (RBCs). The RBCs were resuspended in saline to a 2% concentration and co-cultured with ultrapure water (positive control), PBS (negative control), different concentration of CuO@HA and different concentration of CuO at 37°C for 30 min, respectively. Then the samples were centrifuged at 3000 rpm for 10 min and the absorbance of supernatant at 540 nm were measured by microplate reader. The hemolysis rate was calculated by the formula:

$$\text{Hemolysis rate} = \frac{A_{\text{sample}}\text{-}A_{\text{negative}}}{A_{\text{positive}}\text{-}A_{\text{negative}}} \times 100\%$$

2.8. Intracellular ROS generation

4T1 cells or B16 cells were implanted into confocal dishes and incubated with PBS or CuO@HA NPs (40 μg/mL) for 12 h. Then, the cells co-cultured with DCFH-DA (10 mM) probe for 30 min and then stained with Hoechst 33342 for 10 min. The fluorescence was determined by confocal microscopy.

2.9. Animals

Female BALB/c mice aged 6-8 weeks were purchased from Pengyue Laboratory Animal Breeding Technology Co., Ltd. (Jinan, China) and housed in groups under specific-pathogen-free conditions at 25 °C and 40% relative humidity. All animal experiments were performed with ethical compliance and approved by the Committee

on Animal Welfare of Shandong Provincial Hospital Affiliated to Shandong First Medical University (approval reference NSFC:NO.2023-446).

2.10. In vivo tumor inhibition study

One million B16 or 4T1 cells were injected subcutaneously to the right side of BALB/c mice. After one week, the mice were divided into 2 groups randomly and injected intravenously with PBS or CuO@HA (200 μg) twice for one week interval, respectively. Tumor volumes and mice weights in each mouse were recorded every 2 days. Tumor volume was calculated using the formula $V=0.5\times L\times S^2$, in which L was the largest diameter and S was the smallest diameter of the tumor. 14 days after the first nanoparticles injection, the tumor tissue were collected for photographing, weighing, H&E staining, Ki67 staining and TUNEL staining.

2.11. In vivo biodistribution

4T1 tumor-bearing mice were injected with CuO@HA or CuO intravenously for 0h, 12h and 24h. Then, the heart, liver, spleen, lung, kidney and tumor tissue were collected and digested with HNO_3 and H_2O_2. The Cu concentration was detected by ICP-MS.

2.12. In vivo biosafety study

To assessment the biosafety of the nanoparticles, the BALB/c mice were intravenously injected with PBS or CuO@HA. At day 7, the blood from mice were collected for ALT, AST, BUN and CRE analysis and the major organs (heart, liver, spleen, lung and kidney) were collected for H&E staining.

2.13. Western blot analysis

4T1 cells was cultured with PBS or CuO@HA for 12 h, the cells were then collected and proteins were extracted with RIPA Lysis Buffer IV (Sangon). The protein lysates were then separated with sodium dodecyl sulfate-polyacrylamide gel electrophoresis (SDS-PAGE), transferred onto polyvinylidene difluoride (PVDF) membranes, and western blotting was carried out using a commercial kit (Sangon Biotech Co., Ltd). The PVDF membranes were incubated with primary antibodies (diluted 1:1,000) against CHDH or β-actin and then with a horseradish-peroxidase-conjugated secondary antibody (1:500 dilution). β-actin was used as a loading control.

2.14. RT-PCR analysis

4T1 cells was cultured with PBS or CuO@HA for 12 h. The RNeasy Mini Kit was used to extract RNA according to the manufacturer's instructions. Reverse transcription and qPCR were performed using the AMV First Strand cDNA Synthesis Kit and 2 × SG Fast qPCR Master Mix (High Rox) according to the manufacturer's instructions, respectively.

2.15. Statistical analysis

Student's *t* test was used to compare the means of two groups. $P < 0.05$ was defined as statistically significant. *$p < 0.05$, **$p < 0.01$, and ***$p < 0.001$.

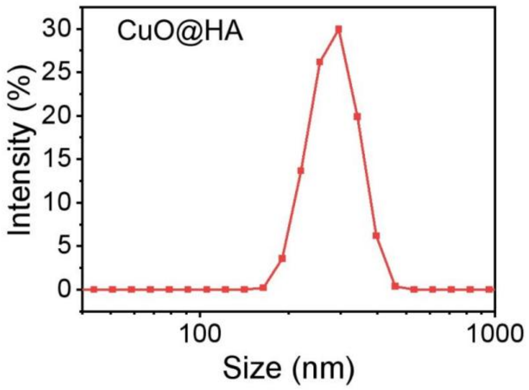

Figure S1. DLS of CuO@HA NPs.

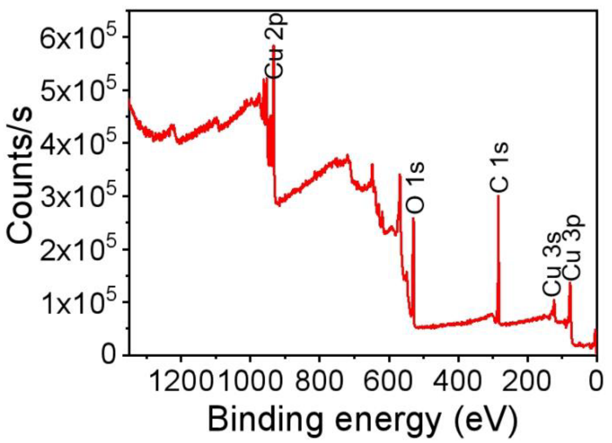

Figure S2. XPS of CuO@HA.

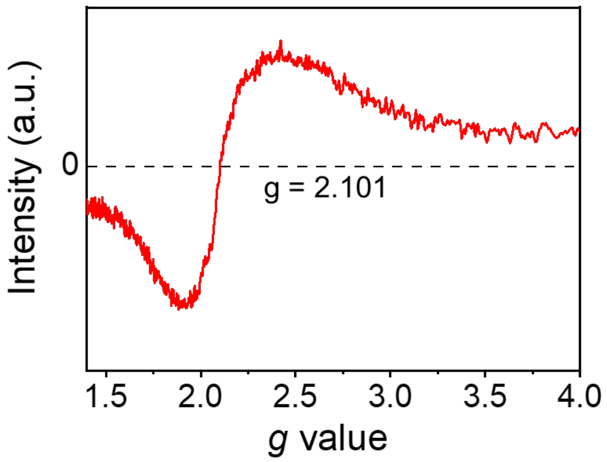

Figure S3. ESR of CuO@HA.

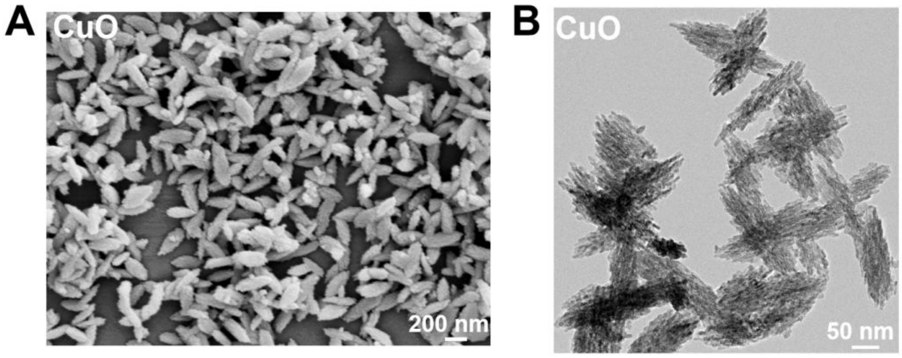

Figure S4. (**A**) SEM and (**B**) TEM of CuO NPs.

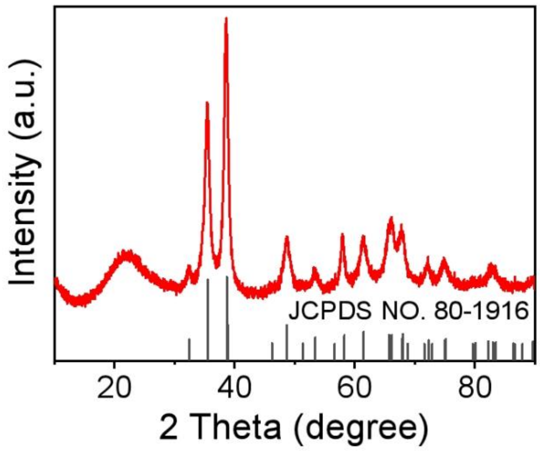

Figure S5. XRD of CuO NPs.

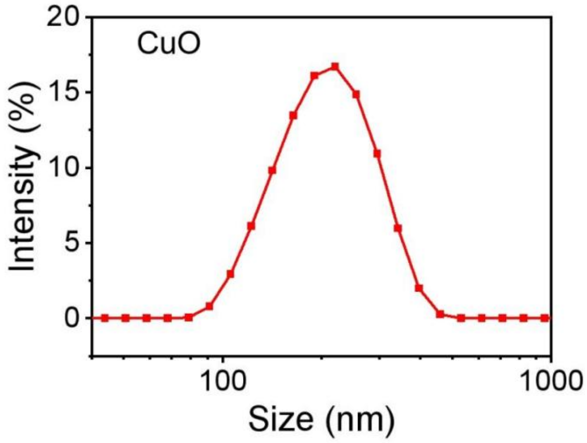

Figure S6. DLS of CuO NPs.

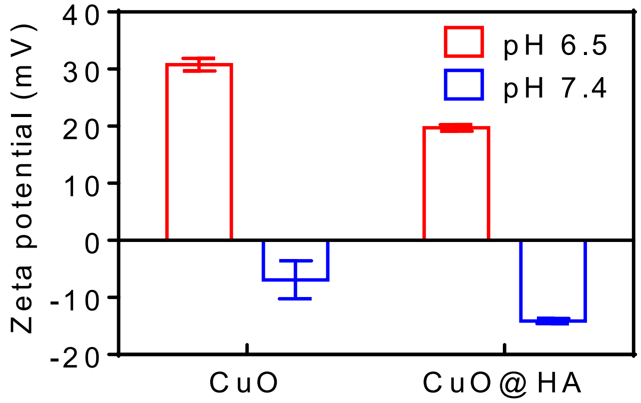

Figure S7. Zeta potential of CuO and CuO@HA in PBS with pH 6.5 and pH 7.4. Data are presented as the mean ± SD (n = 3).

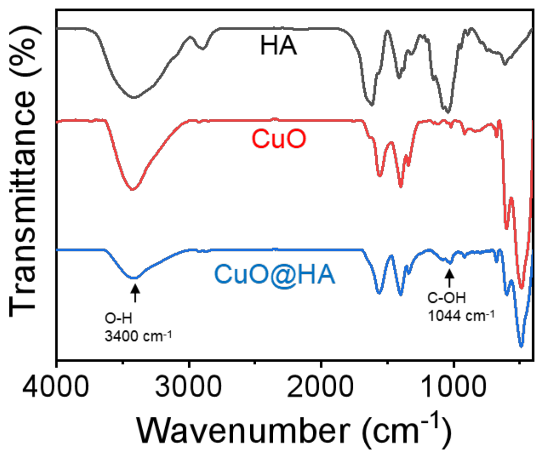

Figure S8. FT-IR spectra of HA, CuO NPs and CuO@HA NPs.

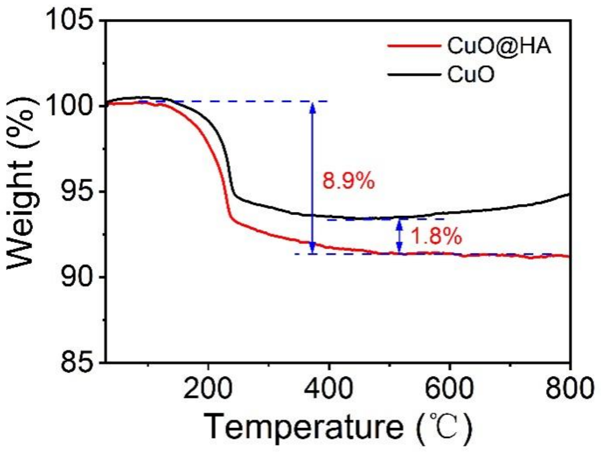

Figure S9. TGA analysis curves of CuO@HA and CuO.

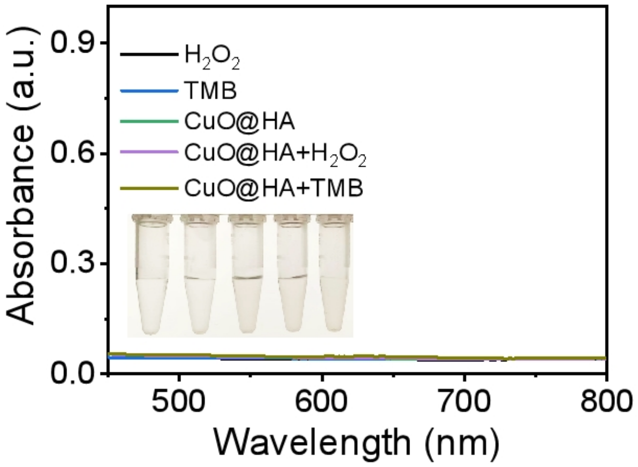

Figure S10. UV-vis spectra of TMB under different conditions (inset: the photo of TMB color with different treatments).

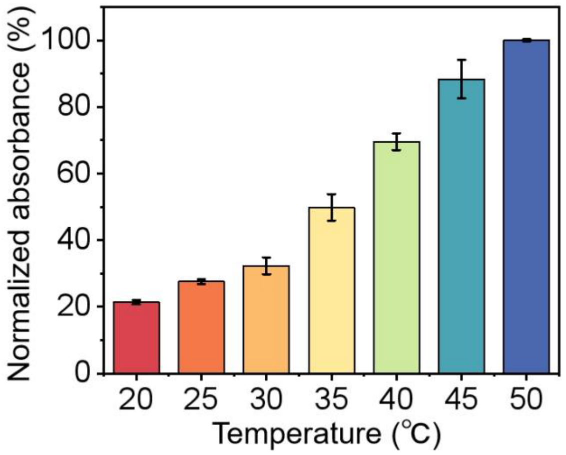

Figure S11. The effect of temperature on the absorbance of TMB chromogenic reaction. Data are presented as the mean ± SD ($n = 3$).

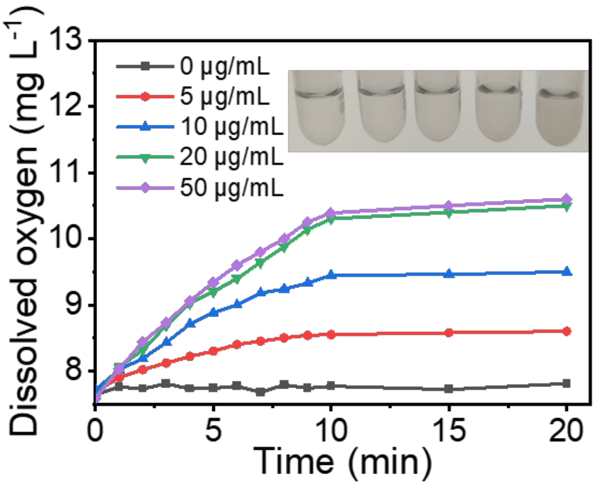

Figure S12. The O_2 generation of different concentration of Cu@HA (0, 5, 10, 20, 50 μg/mL) reacted with H_2O_2 (5 mM) in PBS (pH 6.5) for different time. The insert is the corresponding photograph at 20 min.

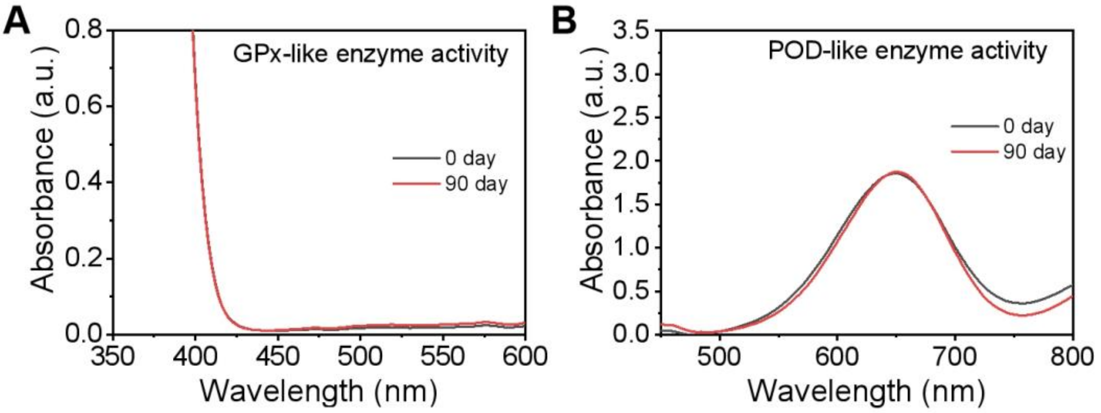

Figure S13. The (**A**) GSHOx-like enzyme activity and (**B**) POD-like enzyme activity of CuO@HA stored for 0 day or 90 day.

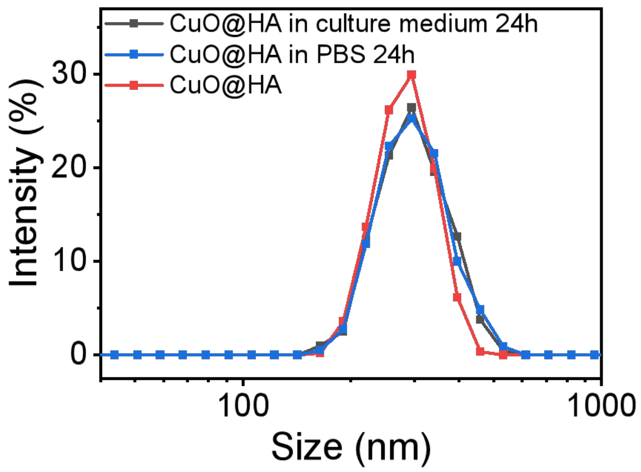

Figure S14. The DLS of CuO@HA, CuO@HA suspended in PBS for 24h and CuO@HA suspended in RPMI-1640 culture medium for 24h.

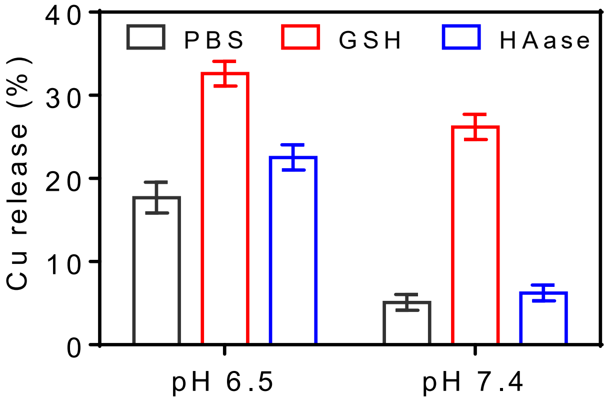

Figure S15. The release of Cu from CuO@HA in different medium. Data are presented as the mean ± SD ($n = 3$).

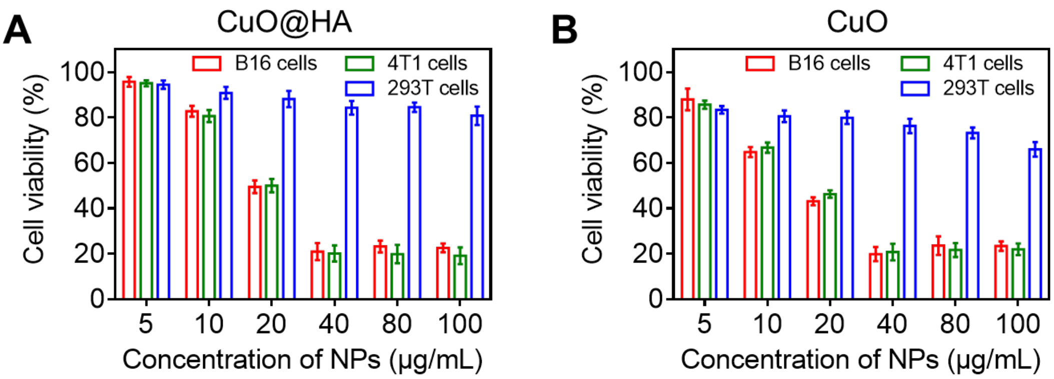

Figure S16. The viabilities of B16, 4T1, and 293T cells treated with different concentrations of (A) CuO@HA or (B) CuO for 12 h. Data are presented as the mean ± SD ($n = 3$).

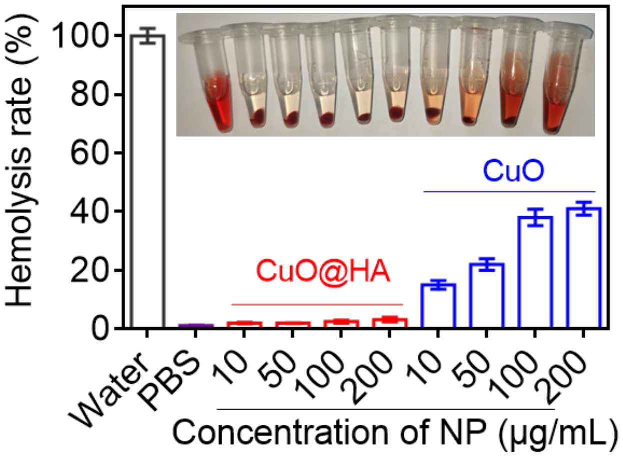

188

189 **Figure S17**. Hemolysis rate of water (positive control), PBS (negative control),
190 different concentration (10, 50, 100, 200 μg/mL) of CuO@HA or CuO. The inset is the
191 photograph of the corresponding samples after centrifugation. Data are presented as the
192 mean ± SD (n = 3).

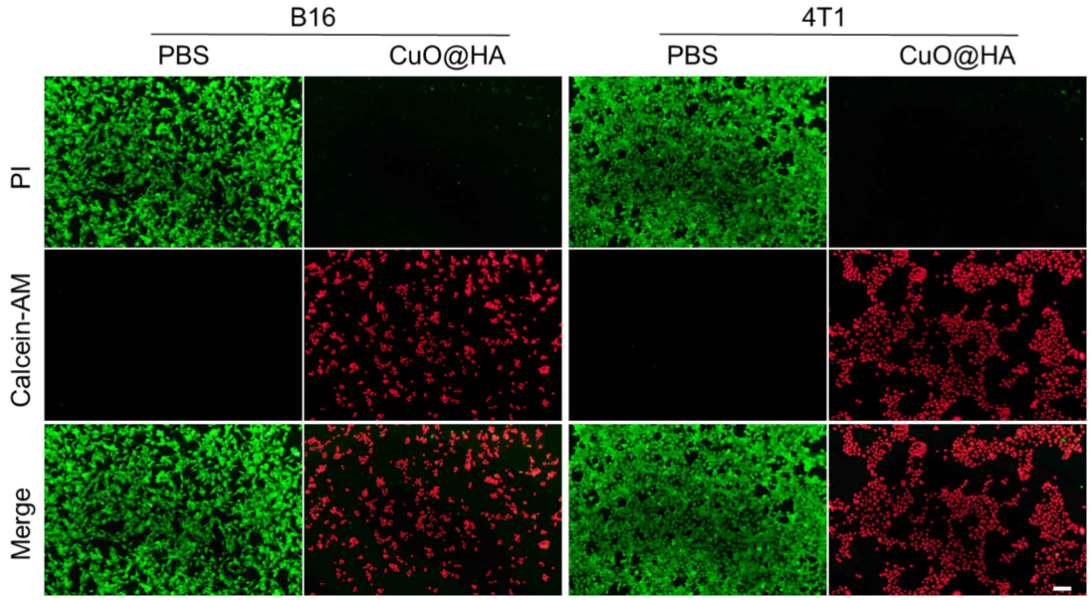

Figure S18. FITC/PI staining of B16 and 4T1 cells cultured with PBS or 40 µg/mL CuO@HA for 12 h. Scale bar, 20 µm .

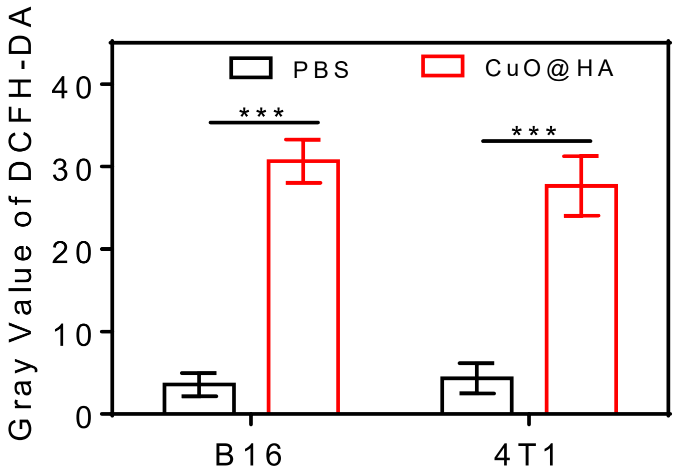

Figure S19. The gray value statistics of DCFH-DA from the corresponding confocal images in Figure 4B. Data are presented as the mean ± SD ($n = 3$). *$p < 0.05$, **$p < 0.01$, and ***$p < 0.001$.

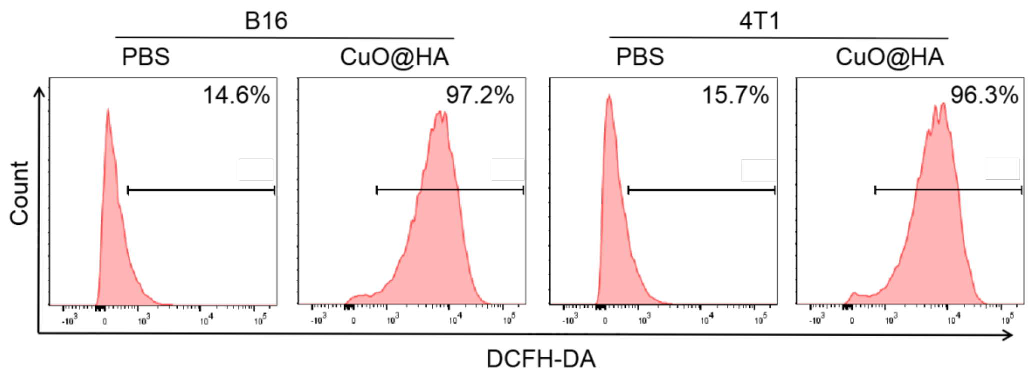

Figure S20. Flow cytometric analysis of ROS levels in B16 and 4T1 cells cultured with PBS or 40 μg/mL CuO@HA for 12 h.

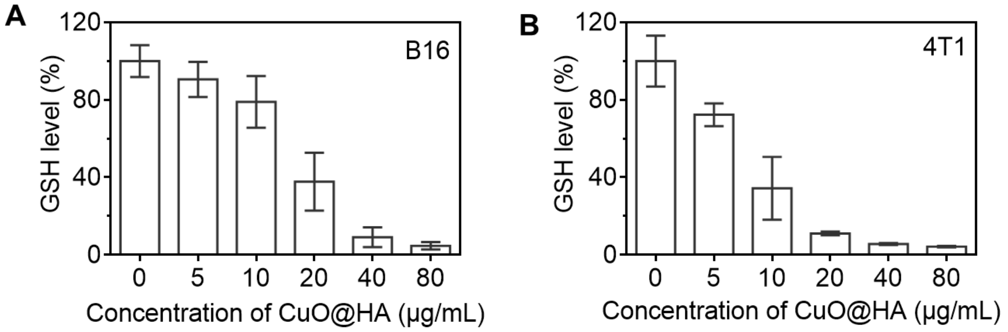

Figure S21. The GSH concentration in (**A**) B16 and (**B**) 4T1 cells treated with different concentration of CuO@HA for 25 min. Data are presented as the mean ± SD ($n = 3$).

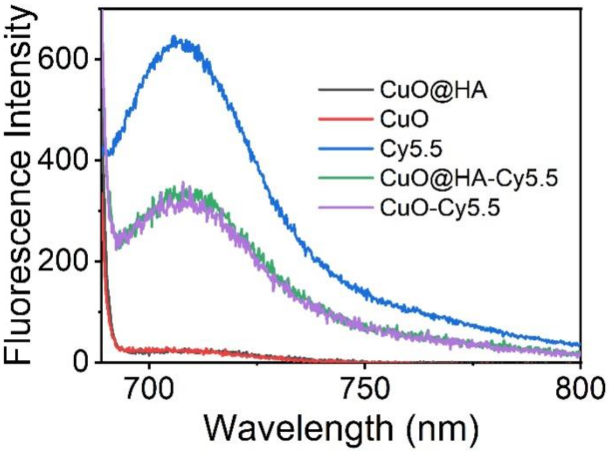

Figure 22. Fluorescence scan of CuO@HA, CuO, Cy5.5, CuO@HA-Cy5.5 and CuO-Cy5.5 under the 680 nm excitation.

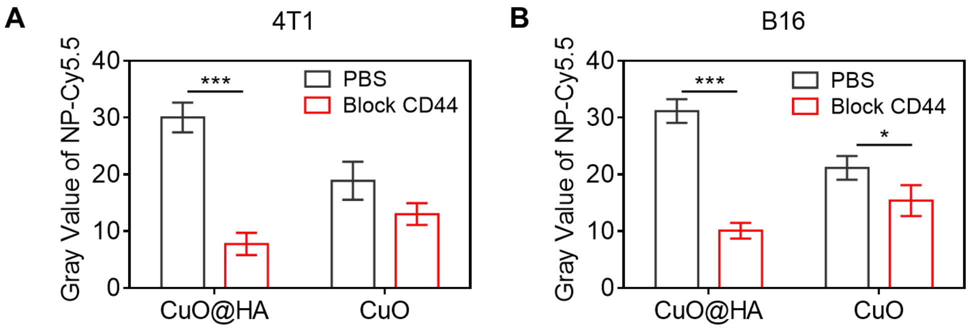

Figure S23. The gray value of nanoparticles-Cy5.5 from the corresponding confocal images in **Figure 4C**. Data are presented as the mean ± SD ($n = 3$). *p < 0.05, **p < 0.01, and ***p < 0.001.

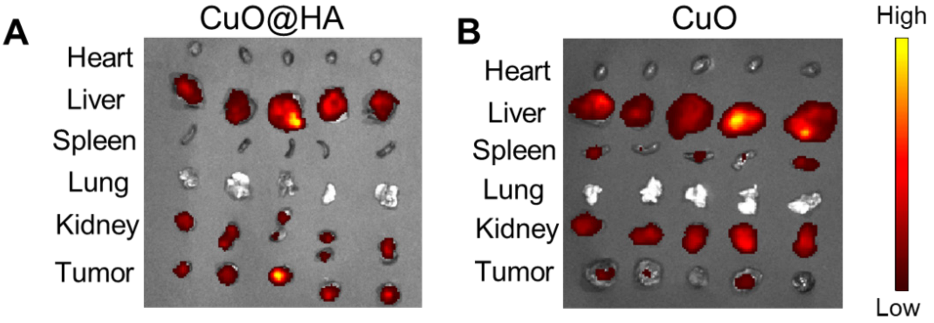

Figure S24. Fluorescence images of tumors and organs dissected from B16 tumor-bearing mice 24h after injection with CuO@HA-Cy5.5 or CuO-Cy5.5.

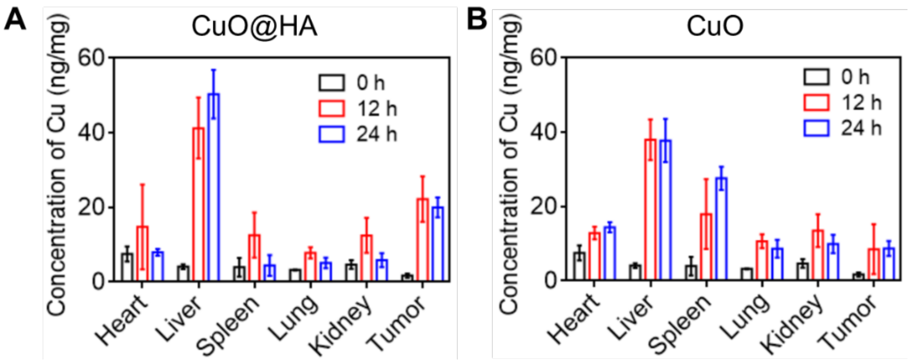

Figure S25. Cu concentration in mouse heart, liver, spleen, lung, kidney and tumor were detected by ICP-MS after intravenously injected with (**A**) CuO@HA or (**B**) CuO for 0 h, 12 h and 24 h. Data are presented as mean ± SD ($n = 5$).

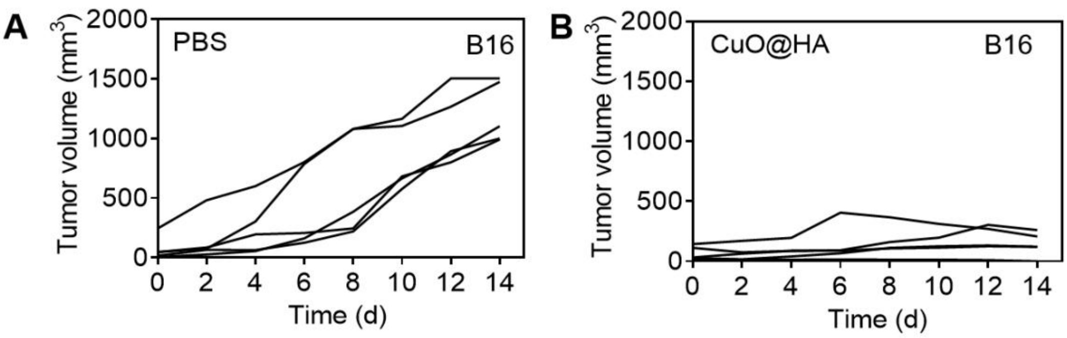

Figure S26. Tumor growth curves of B16 tumor-bearing mice during treated with (**A**) PBS or (**B**) CuO@HA NPs.

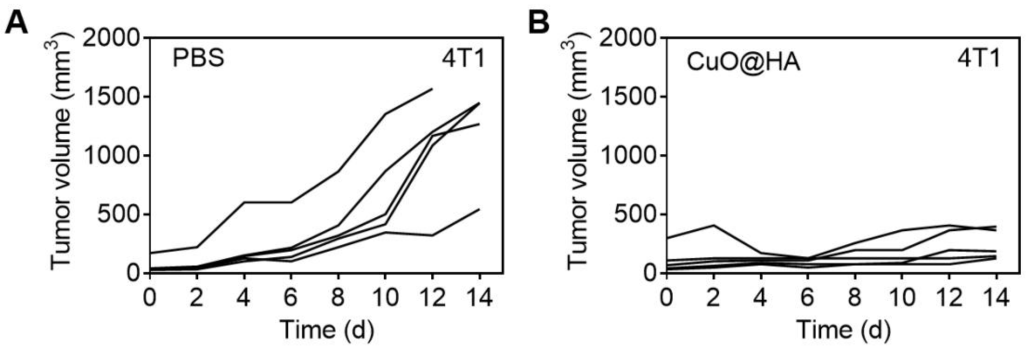

Figure S27. Tumor growth curves of 4T1 tumor-bearing mice during treated with (**A**) PBS or (**B**) CuO@HA NPs.

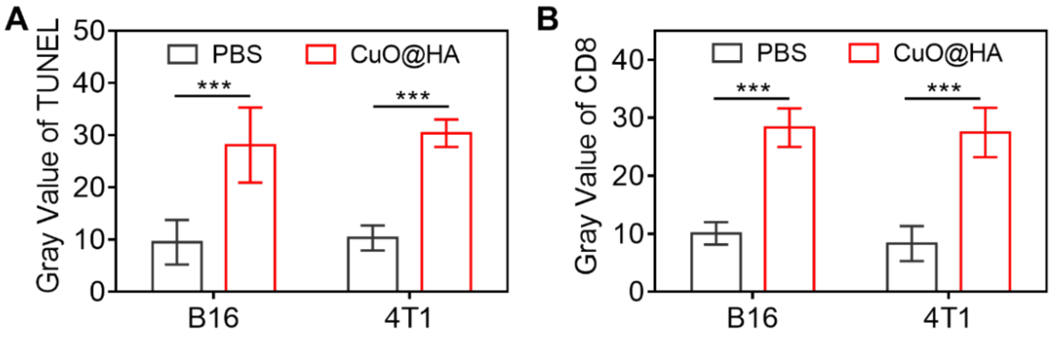

Figure S28. The gray value statistic of (**B**) TUNEL and (**C**) CD8 in **Figure 5I**. Data are presented as the mean ± SD ($n = 3$). *$p < 0.05$, **$p < 0.01$, and ***$p < 0.001$.

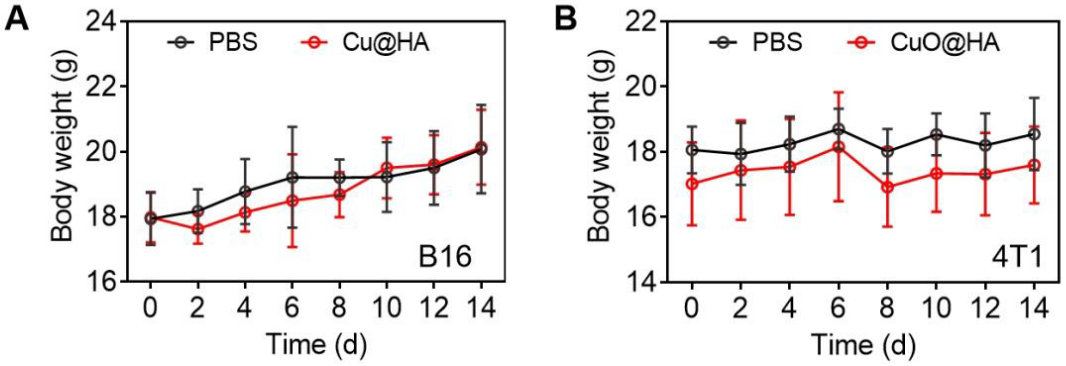

Figure S29. The body weight change curves of (**A**) B16 tumor-bearing mice and (**B**) 4T1 tumor-bearing mice during therapy. Data are presented as the mean ± SD ($n = 5$).

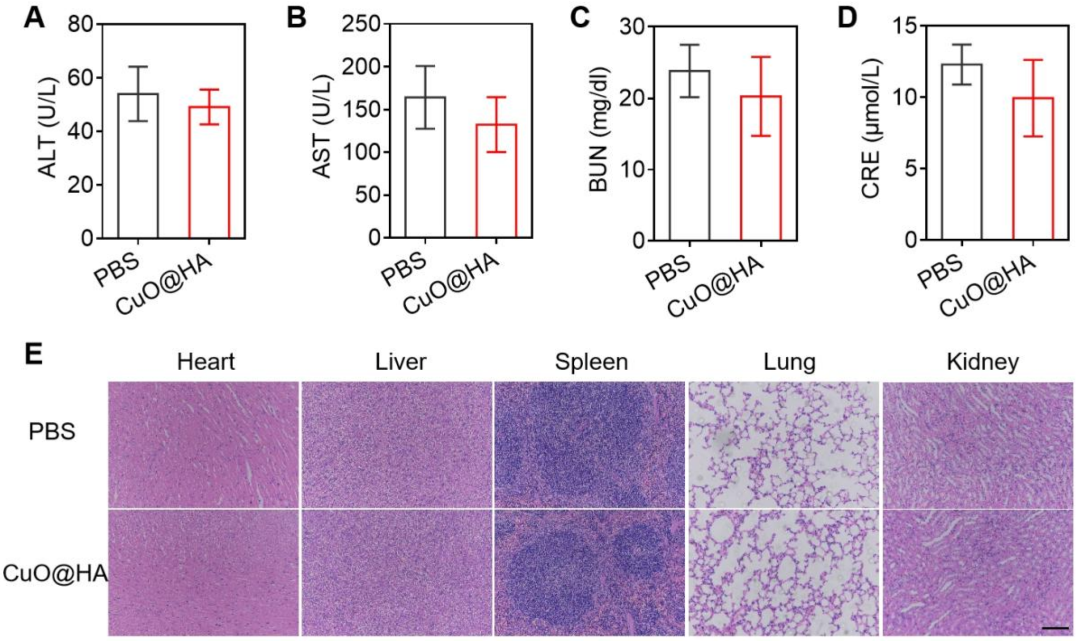

Figure S30. (**A**) Alanine transaminase (ALT) levels, (**B**) aspartate transaminase (AST) levels, (**C**) blood urea nitrogen (BUN) levels, and (**D**) creatinine (CRE) levels in mice after intravenous injection of PBS or CuO@HA for 7 days. (**E**) H&E staining of the heart, liver, spleen, lung, and kidney of mice after intravenous injection of PBS or CuO@HA. Scale bars: 100 μm. Data are presented as the mean ± SD (n = 3).

238 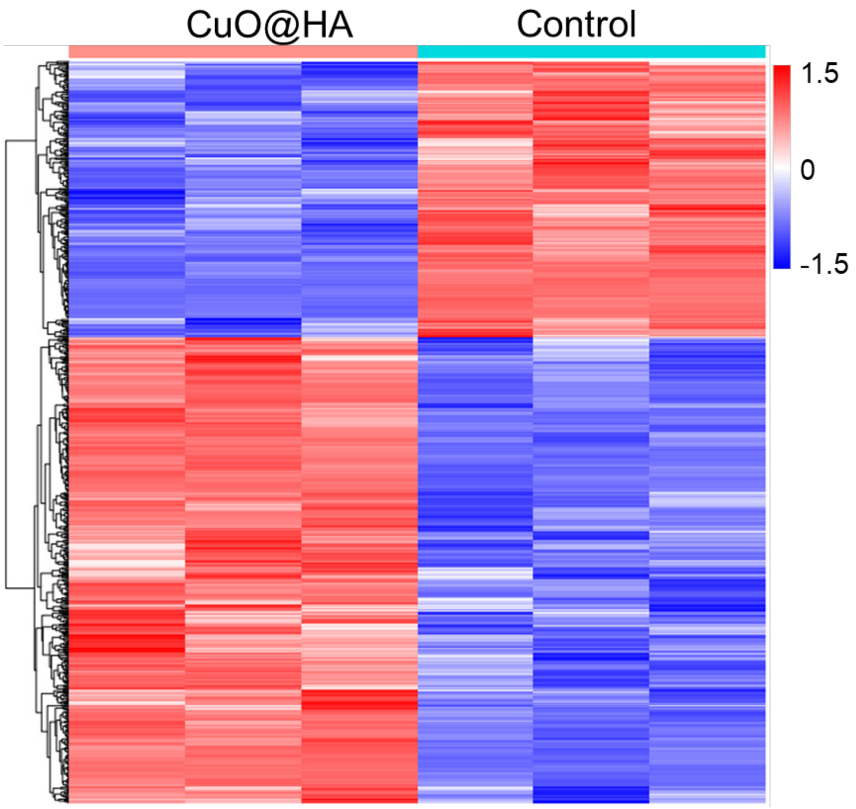

239 **Figure S31**. Heatmap of differentially expressed metabolism.

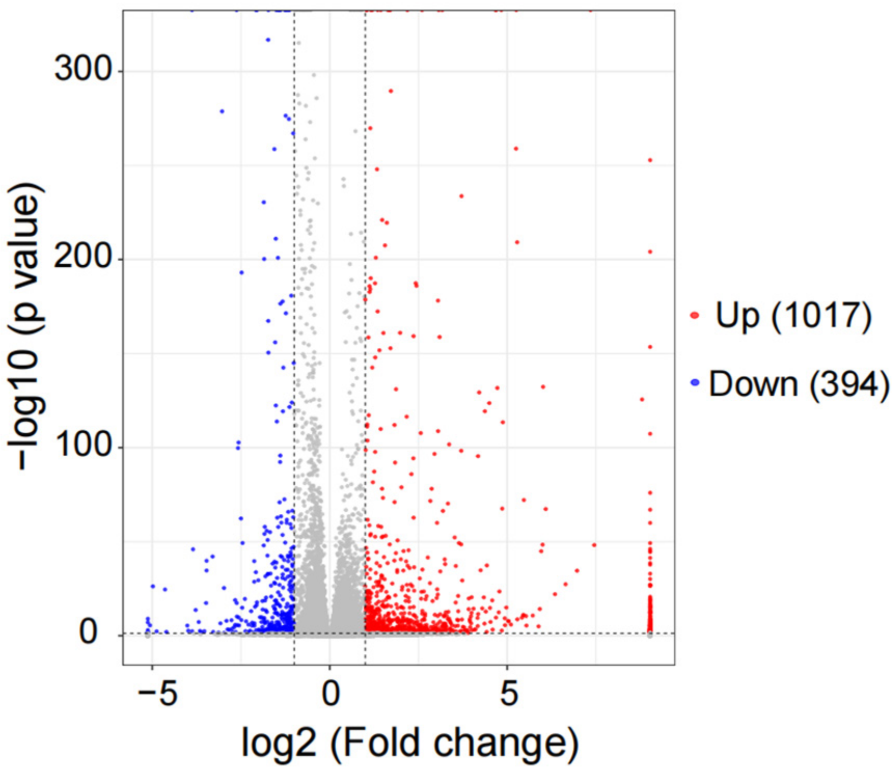

Figure S32. Volcano plot of upregulated and downregulated genes. $p < 0.05$, FC > 2.

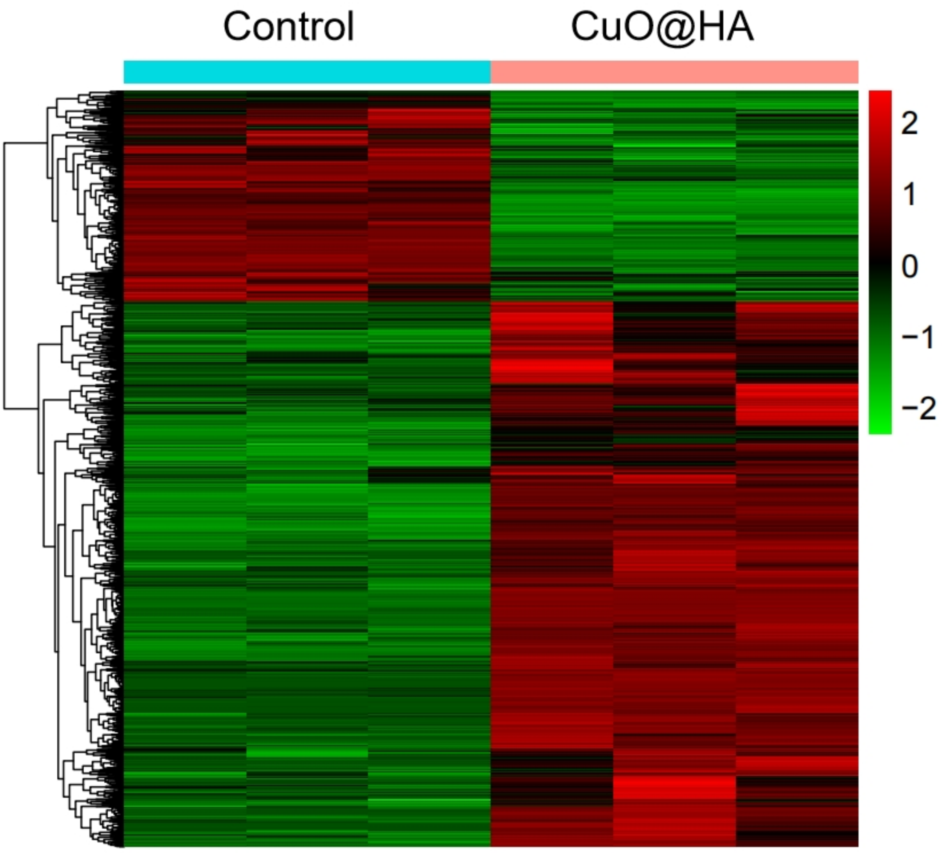

Figure S33. Heatmap of differentially expressed gene.

Supplementary Table 1. Quantitative RT-PCR primer sequence.

**Gene name**	**Sequence**
chdh	Forward: GGAGCCATCAACTCTCCACAG
	Reverse: CAGACCTTCCGCAGAGGCTT
β-actin	Forward: GTGCTATGTTGCTCTAGACTTCG
	Reverse: ATGCCACAGGATTCCATACC
